# A Genome-wide Association Study of Susceptibility to Upper Urinary Tract Infections

**DOI:** 10.1093/infdis/jiae231

**Published:** 2024-05-07

**Authors:** Helene M Flatby, Anuradha Ravi, Kristin V Liyanarachi, Jan E Afset, Humaira Rasheed, Ben M Brumpton, Kristian Hveem, Bjørn O Åsvold, Andrew T DeWan, Erik Solligård, Jan K Damås, Tormod Rogne

**Affiliations:** Mid-Norway Centre for Sepsis Research, Department of Circulation and Medical Imaging, Norwegian University of Science and Technology; Clinic of Anaesthesia and Intensive Care; Mid-Norway Centre for Sepsis Research, Department of Circulation and Medical Imaging, Norwegian University of Science and Technology; Department of Medical Genetics; Mid-Norway Centre for Sepsis Research, Department of Circulation and Medical Imaging, Norwegian University of Science and Technology; Department of Infectious Diseases, St Olavs Hospital, Trondheim University Hospital; Mid-Norway Centre for Sepsis Research, Department of Circulation and Medical Imaging, Norwegian University of Science and Technology; Department of Clinical and Molecular Medicine, Norwegian University of Science and Technology; Department of Medical Microbiology, St Olavs Hospital, Trondheim University Hospital; K. G. Jebsen Center for Genetic Epidemiology, Department of Public Health and Nursing, Norwegian University of Science and Technology; Clinic of Medicine, St Olavs Hospital, Trondheim University Hospital, Trondheim; K. G. Jebsen Center for Genetic Epidemiology, Department of Public Health and Nursing, Norwegian University of Science and Technology; Clinic of Medicine, St Olavs Hospital, Trondheim University Hospital, Trondheim; The Trøndelag Health Study Research Center, Department of Public Health and Nursing, Norwegian University of Science and Technology, Levanger; K. G. Jebsen Center for Genetic Epidemiology, Department of Public Health and Nursing, Norwegian University of Science and Technology; Department of Research, Innovation, and Education; K. G. Jebsen Center for Genetic Epidemiology, Department of Public Health and Nursing, Norwegian University of Science and Technology; The Trøndelag Health Study Research Center, Department of Public Health and Nursing, Norwegian University of Science and Technology, Levanger; Department of Endocrinology, Clinic of Medicine, St Olavs Hospital, Trondheim University Hospital, Trondheim, Norway; Mid-Norway Centre for Sepsis Research, Department of Circulation and Medical Imaging, Norwegian University of Science and Technology; Department of Chronic Disease Epidemiology and Center for Perinatal, Pediatric and Environmental Epidemiology, Yale School of Public Health, New Haven, Connecticut; Mid-Norway Centre for Sepsis Research, Department of Circulation and Medical Imaging, Norwegian University of Science and Technology; Department of Innovation, Education and Health Sciences, Helse Møre og Romsdal Hospital Trust, Ålesund, Norway; Mid-Norway Centre for Sepsis Research, Department of Circulation and Medical Imaging, Norwegian University of Science and Technology; Department of Infectious Diseases, St Olavs Hospital, Trondheim University Hospital; Centre of Molecular Inflammation Research, Department of Clinical and Molecular Medicine, Norwegian University of Science and Technology, Trondheim, Norway; Mid-Norway Centre for Sepsis Research, Department of Circulation and Medical Imaging, Norwegian University of Science and Technology; Department of Chronic Disease Epidemiology and Center for Perinatal, Pediatric and Environmental Epidemiology, Yale School of Public Health, New Haven, Connecticut

**Keywords:** upper urinary tract infection, pyelonephritis, genome-wide association study, Mendelian randomization, smoking

## Abstract

**Background:**

Our goal was to identify genetic and modifiable risk factors for upper urinary tract infections (UTIs).

**Methods:**

We used data from UK Biobank, the Trøndelag Health Study, and the Michigan Genomics Initiative to conduct genome-wide association studies and sex-stratified analyses on upper UTI. Mendelian randomization (MR) analyses were conducted to examine potential causal relationships between cardiometabolic risk factors and upper UTIs.

**Results:**

One genome-wide significant (*P* ≤ 5E-08) locus was associated with the susceptibility to upper UTI, located near *TSN* in the female-only analysis. Additionally, we identified suggestive (*P* ≤ 5E-06) loci near *DNAI3* for females, *SCAMP1−AS1* for males, and near *TSN*, *LINC00603*, and *HLA-DQA2* for both sexes. In MR analyses, higher genetically predicted lifetime smoking scores were associated with an increased risk of developing upper UTI for females and both sexes (odds ratio [OR], 4.84, *P* = 4.50E-06 and OR, 2.79, *P* = 3.02E-05, respectively).

**Conclusions:**

We found that genetic variants near *TSN* was associated with the risk of upper UTIs among females. In addition, we found several genetic loci with suggestive associations with the risk of upper UTIs. Finally, MR analyses found smoking to be a potential causal risk factor for upper UTIs.

## Teaser

Genome-wide association studies identify genome-wide significant variants near the *TSN* locus associated with increased risk of upper urinary tract infection (UTI) for females. Suggestive loci were identified in the analysis of females, males, and both sexes. Mendelian randomization analysis links smoking to increased risk of upper UTI.

Urinary tract infections (UTIs) are common bacterial infections worldwide, with a significant clinical and economic burden [1]. UTIs typically affect the lower urinary tract (eg, bladder [cystitis]), but the infection may ascend upward in the urinary tract to cause upper UTIs—ureteritis affecting the ureters, or pyelonephritis affecting the kidneys—which are less common but more severe [2]. Therefore, differentiating between lower and upper UTIs is important in terms of etiology and outcomes. As is the case for other infections, the risk of acquiring upper UTIs involves a complex interplay between host genetic factors, the virulence of the pathogen, and individual patient clinical characteristics such as anatomy and underlying risk factors [2].

While many complex diseases have clear links to genetic variants, the genetic susceptibility to upper UTIs remains largely unknown. Previous family-based studies have found that different genetic mechanisms may influence the susceptibility of lower and upper UTIs [3–5]. Among these, 1 family study found an increased frequency of acute pyelonephritis (ie, upper UTI) in family members of acute pyelonephritis–prone children, while no differences were observed for acute cystitis (ie, lower UTI) [3]. However, few genome-wide association studies (GWASs) have been conducted on upper UTIs. As of today, only 1 published GWAS has been conducted on pyelonephritis, where 1 locus on chromosome 14 close to *LINC02302* was found to be strongly associated with pyelonephritis using data from BioBank Japan [6]. There is, therefore, a need for GWASs conducted on upper UTIs to identify genes and pathways associated with susceptibility to upper UTIs, which may be important for risk stratification and prevention and may also lead to new insight into therapeutic targets [7].

In addition to mapping the genetic susceptibility to upper UTIs, identifying modifiable risk factors for upper UTIs is key to prevention, especially considering the increasing antibiotic resistance in uropathogenic bacteria and the demographic shift toward an older population [8]. Traditional epidemiological studies have found that obesity [9] and type 2 diabetes mellitus (T2DM) [10] increase the risk of UTIs in general. However, traditional epidemiological studies may be subject to confounding (eg, from lifestyle factors) and reverse causation. Mendelian randomization (MR) analysis provides an alternative method to assess causality using genetic variants identified through GWAS as instrumental variables. MR exploits nature’s random assortment of alleles from parents to offspring during gamete formation, mimicking a randomized clinical trial. This greatly reduces the likelihood of confounding and reverse causation [11]. By using MR, previous studies have found that genetically predicted high body mass index (BMI) increases susceptibility to UTIs (defined as a combination of upper or lower UTIs) [12, 13]. In contrast to observational studies, an MR study found no causal relationship between T2DM and the risk of UTIs (defined as a combination of upper or lower UTIs) [14].

To investigate the genetic susceptibility of upper UTIs, we conducted a GWAS of independent cohorts of subjects of European ancestry. To examine the potential causal relationships between cardiometabolic risk factors and upper UTIs, we performed 2-sample MR analyses.

## METHODS

### Study Population Used in Genome-wide Association Analyses

We used individual-level data from the UK Biobank and HUNT for the genome-wide association analyses and summary-level data from the Michigan Genomics Initiative (MGI). All subjects were of European ancestry (Supplementary Text, Cohort’s description).

### Phenotype

In this study, we evaluated the risk of upper UTIs using hospital diagnosis codes of UTIs above the bladder (ie, involving the ureters and kidney). The *International Classification of Diseases*, *Ninth Revision* (*ICD-9*) and *Tenth Revision* (*ICD-10*) codes retrieved from hospital records were used to identify cases and controls. For each cohort, cases and controls were drawn from the same population. Cases were participants with at least 1 inpatient *ICD-9/10* code as a primary or secondary code for upper UTIs, while participants with no *ICD-9/10* (primary or secondary diagnosis) for upper UTIs were used as controls. The same *ICD-9/10* codes were used to identify cases for all analyses: *ICD-9* code 590 (infections of the kidney) and *ICD-10* codes N10 (acute pyelonephritis) and N12 (tubelo-interstitial nephritis, not specified as acute or chronic). Field IDs used for UK Biobank are presented in Supplementary Table 1.

### Genome-wide Association Analyses

The scalable and accurate implementation of generalized mixed models (SAIGE) was used to perform genome-wide association analyses (Supplementary Text, Genome-wide association analyses) [15]. Because of potential sex differences in the genetic susceptibility to upper UTI [2], we conducted separate GWAS analyses for females and males in the UK Biobank and HUNT. This was not possible with summary-level data available from MGI. In addition, we conducted analyses that considered both sexes, which included UK Biobank, HUNT, and MGI. Meta-analyses were conducted in METAL (version 2011-03-25) [16] using fixed effect and weighted by the standard errors. We applied genomic control correction in the meta-analysis to account for population stratification or unaccounted relatedness for each study. *I^2^* and Cochran *Q*-test for heterogeneity [17], implemented in METAL [16], were calculated to assess heterogeneity of single-nucleotide polymorphism (SNP) outcome associations between the included datasets [18]. A total of 8 623 402 SNPs were identified for the female-only analysis, 8 614 190 SNPs for the male-only analysis, and 10 235 765 SNPs for the analysis of both sexes.

If not otherwise specified, results are presented from the meta-analysis.

### Replication of Top Hits From Previous GWASs on UTIs and Phenome-wide Association Analysis

We examined if we could replicate previously reported associations from 3 studies of lower and/or upper UTIs [6, 19, 20]. Information about the cohorts used, phenotype definition, number of cases and controls, and the top hit for each of the 3 studies are shown in Supplementary Tables 2 and 3. We used the meta-analysis results from the analysis of both sexes as the main replication study. For missing SNPs, we used the LDproxy tool from the National Cancer Institute LDlink [21] to find potential linkage disequilibrium (LD) proxy SNPs in European population by applying a threshold of *R^2^* > 0.9. Associations were considered replicated if the β-coefficient was in the same direction and the *P* < .05.

We also performed phenome-wide association (PheWAS) analyses to examine if the genetic variants identified in our study were linked to other phenotypes. PheWAS analyses were conducted through the Open Targets platform (genetics.opentargets.org, accessed 5 April 2023) [22] for genome-wide significant (*P* ≤ 5E-08) or suggestive variants (*P* ≤ 1E-06) identified in the analyses of females only, males only, and both sexes.

### Mendelian Randomization Analysis

Mendelian randomization is a study design used to explore the association between an exposure and an outcome, where selected genetic variants identified from GWASs are used as instrumental variables for the exposure of interest. Three key assumptions must be met for an instrumental variable to be valid: There must be a robust association between the instrumental variable and the exposure, the instrumental variable should have no shared cause with the outcome, and only affect the outcome through the risk factor [23].

We only included independent SNPs (*r^2^* < 0.001 within 10 000 kb windows) strongly associated (*P* ≤ 5E-08) with each cardiometabolic trait of interest. The traits evaluated were chosen based on results from previous traditional observational studies and/or MR studies. BMI has been found to be associated with UTI [12, 13], while conflicting results have been reported for T2DM [10, 14, 24, 25]. Smoking, low-density lipoprotein cholesterol (LDL-C), and systolic blood pressure (SBP) have not previously been linked to UTI. However, smoking was included because it is a known risk factor for several infections [26], and LDL-C was included due to being an important modulator of the immune response, which may affect the risk of infection [27]. A recent MR study found an association between genetically predicted blood pressure and subsequent alteration in lymphocyte counts [28]. Therefore, a total of 5 traits related to cardiometabolic risk factors were included as exposures: BMI [29], lifetime smoking index (referred to as smoking) [30], LDL-C [31], SBP [32], and T2DM [33] (details about the GWASs of each exposure are presented in the Supplementary Text and Supplementary Tables 4 and 5). The meta-analysis results from the female-only and male-only analyses and the analysis of both sexes were used as the main outcomes for the MR analyses. For the analysis of both sexes, we also performed MR analyses separately for UK Biobank, HUNT, and the MGI. To avoid biases due to population stratification, we only evaluated participants of European ancestry in the GWASs and MR analyses.

The main analysis was performed using the inverse variance–weighted method, combining the causal effect estimates from each genetic instrument [34]. Several sensitivity analyses were performed, including MR Egger, MR Egger intercept test, weighted median, simple mode, and weighted mode, which estimate the causal effect under different assumptions about horizontal pleiotropy [11]. Between-SNP heterogeneity of the causal estimate was evaluated using Cochran *Q* statistical test [35]. To account for multiple testing of 5 exposures, we set the threshold for statistical significance to *P* < .01 (equal to 0.05/5), while *P* < .05 was considered nominally significant.

### Software

GWAS and MR analyses were carried out using R (version 3.6.1 and 4.0.0, respectively), METAL (version 2011-03-25), PLINK (version 1.9), and BOLT-LMM (version 2.3.4) software. MR analyses were conducted using the TwoSampleMR R package (version 0.5.4) [35].

### Ethical Approval

The HUNT study was approved by the Regional Committee for Medical Research, Health Region IV, in Norway. Additionally, this project is regulated in accordance with the Norwegian Social Science Data Services. The UK Biobank study has ethical approval from the Northwest Multi-Center Research Ethics Committee, and the Research Tissue Bank covers individual project approval. The University of Michigan Medical School Institutional Review Board (IDs HUM00071298, HUM00148297, HUM00099197, HUM00097962, and HUM00106315) reviewed and approved MGI study participants’ consent forms.

## RESULTS

### Study Population Characteristics

The female-only analysis included 1831 cases and 255 541 controls, while the male-only analysis included 896 cases and 219 040 controls. The analysis of both sexes totaled 3873 cases and 512 608 controls. The baseline characteristics for cases and controls in the UK Biobank and HUNT are shown in Table 1. Since we only used summary statistics, this information was unavailable for the 1146 cases and 38 027 controls included for MGI. For more information regarding MGI, please see [36].

**Table 1.** Background Characteristics for UK Biobank and the Trøndelag Health Study

Study	Sex	Group	Age, y^a^	Ever-smoker^b^	Diabetes (Self-reported)^b^	BMI^c^, kg/m^2^	LDL-C, mmol/L^c^	Systolic BP, mm Hg^c^
UK Biobank	Females	Cases (n = 1163)	56 (49–63)	688 (59.1)	98 (8.4)	27.9 (5.7)	3.5 (0.9)	139.2 (19.8)
		Controls (n = 219 446)	57 (51–63)	121 995 (55.6)	7769 (3.5)	27.0 (5.1)	3.7 (0.9)	137.6 (20.2)
		All (n = 220 609)	57 (51–63)	122 683 (55.6)	7867 (3.6)	27.0 (5.1)	3.6 (0.9)	137.6 (20.2)
	Males	Cases (n = 418)	59 (54–65)	297 (71.1)	72 (17.2)	28.8 (5.1)	3.3 (0.9)	143.4 (17.8)
		Controls (n = 186 861)	57 (51–64)	121 834 (65.2)	12 522 (6.7)	27.8 (4.2)	3.4 (0.9)	143.2 (18.5)
		All (n = 187 279)	57 (51–64)	122 131 (65.2)	12 594 (6.7)	27.8 (4.2)	3.5 (0.9)	143.2 (18.4)
	Both sexes	Cases (n = 1581)	57 (50–63)	985 (62)	170 (11)	28.1 (8.6)	3.5 (0.9)	140.2 (19.6)
		Controls (n = 406 307)	57 (51–63)	243 829 (60)	20 291 (5)	27.4 (4.8)	3.6 (0.9)	139.2 (19.8)
		All (n = 407 888)	57 (51–63)	244 814 (60)	20 461 (5)	27.4 (4.8)	3.6 (0.9)	139.2 (19.8)
HUNT	Females	Cases (n = 688)	56 (37–70)	371 (53.9)	51 (7.4)	26.6 (5.6)	3.7 (1.3)	138.2 (27.9)
		Controls (n = 36 095)	44 (31–59)	19 312 (53.5)	1640 (4.5)	27.0 (6.3)	3.5 (1.1)	131.4 (24.8)
		All (n = 36 783)	45 (31–60)	19 683 (53.5)	1691 (4.6)	26.6 (5.6)	3.6 (1.2)	131.5 (24.8)
	Males	Cases (n = 458)	64 (52–72)	355 (77.5)	53 (11.6)	26.8 (6.3)	3.5 (1.1)	140.8 (29.0)
		Controls (n = 32 179)	45 (32–59)	19 817 (61.6)	1667 (5.2)	27.0 (4.3)	3.4 (1.1)	136.2 (20.6)
		All (n = 32 637)	45 (32–59)	20 172 (61.8)	1720 (5.3)	27.0 (4.3)	3.4 (1.2)	136.2 (20.8)
	Both sexes	Cases (n = 1146)	60 (45–71)	726 (63.4)	104 (9.1)	26.9 (6.3)	3.5 (1.1)	139.2 (28.3)
		Controls (n = 68 274)	43 (32–59)	39 129 (57.3)	3307 (4.8)	26.8 (5.0)	3.6 (1.2)	133.7 (23.0)
		All (n = 69 420)	45 (32–59)	39 855 (57.4)	3411 (4.9)	26.8 (5.0)	3.6 (1.2)	133.8 (23.1)

Abbreviation: BMI, body mass index; BP, blood pressure; HUNT, Trøndelag Health Study; LDL-C, low-density lipoprotein cholesterol.

^a^Data are presented as median (interquartile range).

^b^Data are presented as No. (%).

^c^Data are presented as mean (standard deviation).

### Genome-wide Association Analysis

**Figure 1.**
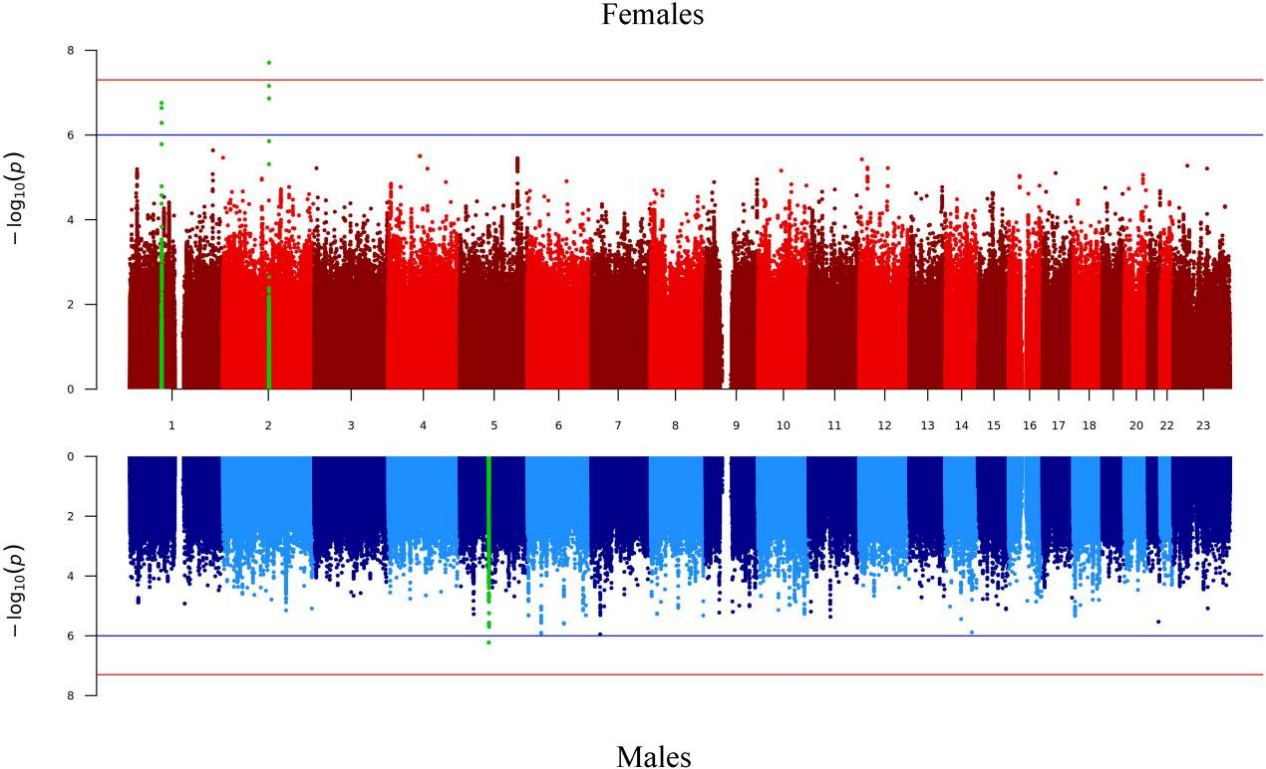
Miami plot of the genome-wide association analysis for the risk of upper urinary tract infection for the sex-stratified meta-analyses. The x-axis shows the genomic position (chromosomes 1–23; where 23 is the X chromosome), and the y-axis represents the negative logarithm (base 10) of the variant *P* value. The blue lines indicates genome-wide suggestive associations (*P* ≤ 1E-06), and the red lines indicates genome-wide significant associations (*P* ≤ 5E-08). The red Manhattan plot indicates the female-only genome-wide association study (GWAS), and the blue Manhattan plot indicates male-only GWAS. Genome-wide significant or suggestive loci in the female- or male-only analysis (±500 kb of lead variant) are highlighted in green.

We observed a genome-wide significant hit in the female-only analysis (Figure 1, Table 2, Supplementary Figure 1). This locus was near *TSN* on chromosome 2, where the presence of the effect allele (C) was associated with an odds ratio (OR) for upper UTI of 2.64 (95% confidence interval [CI], 2.30–2.98; *P* = 1.96E-08). This genetic variant was not associated with upper UTIs among males (OR, 0.96 [95% CI, .50–1.43; *P* =8.73E-01]). We also observed 1 suggestive region near *DNAI3* on chromosome 1 (rs114573015) that was associated with an increased risk of upper UTIs among females (OR, 2.03 [95% CI, 1.76–2.29; *P* = 1.76E-07]).

**Table 2.** Genetic Variants Associated With Upper Urinary Tract Infections in the Female-Only and Male-Only Analysis

Sex	SNP Information					Meta-analysis						UK Biobank				HUNT			
	rsid	Chr	Pos^a^	Closest Gene	EA/OA	EAF	OR (95% CI)	*P* Value	D	*I^2^*	*I^2^ P* Value	EAF	OR (95% CI)	*P* Value	*INFO*	EAF	OR (95% CI)	*P* Value	*R^2^*
Females	rs114573015	1	85603051	*DNAI3*	G/A	0.02	2.03 (1.76–2.29)	1.76E-07	++	0	6.93E-01	0.02	2.09 (1.78–2.40)	2.41E-06	0.95	0.01	1.85 (1.32–2.37)	2.15E-02	0.95
	rs114370000	2	123333053	*TSN*	C/G	0.01	2.64 (2.30–2.98)	1.96E-08	++	0	9.27E-01	0.01	2.63 (2.20–3.05)	7.83E-06	0.88	0.01	2.66 (2.10–3.22)	6.05E-04	0.80
	rs11749979	5	77640190	*SCAMP1- AS1*	C/T	0.39	0.96 (.89–1.03)	2.30E-01	–	0	9.32E-01	0.40	0.96 (.87–1.04)	3.12E-01	0.99	0.39	0.96 (.85–1.07)	5.10E-01	1.00
Males	rs114573015	1	85603051	*DNAI3*	G/A	0.02	1.07 (.68–1.46)	7.50E-01	−+	44	1.82E-01	0.02	0.77 (.29–1.26)	2.93E-01	0.95	0.01	1.34 (.69–2.00)	3.76E-01	0.95
	rs114370000	2	123333053	*TSN*	C/G	0.01	0.96 (.50–1.43)	8.73E-01	−+	0	8.64E-01	0.01	1.00 (.35–1.66)	9.94E-01	0.88	0.01	0.92 (.27–1.58)	8.15E-01	0.80
	rs11749979	5	77640190	*SCAMP1- AS1*	C/T	0.39	1.28 (1.18–1.38)	5.95E-07	++	23	2.55E-01	0.40	1.21 (1.07–1.35)	7.63E-03	0.99	0.39	1.36 (1.22–1.49)	1.23E-05	1

Only variants with *P* ≤ 1E-06 in the meta-analysis for females or males are displayed.

Abbreviations: Chr, chromosome; CI, confidence interval; D, direction of effect; EA, effect allele; EAF, effect allele frequency; *I^2^*, heterogeneity; *INFO*, imputation score from IMPUTE2; OA, other allele; OR, odds ratio; Pos, chromosome position; *R^2^*, imputation score from Minimac3.

^a^Position of each single-nucleotide polymorphism is given along the chromosome Build 37.

There was 1 locus that was suggestively associated with male upper UTIs: rs11749979 near *SCAMP1−AS1* on chromosome 5 was associated with an OR of upper UTIs of 1.28 (95% CI, 1.18–1.38; *P* = 5.95E-07; Figure 1, Table 2, Supplementary Figure 2). The signal was stronger among males compared with females (OR, 0.96 [95% CI, .89–1.03]; *P* = 2.30E-01).

**Figure 2.**
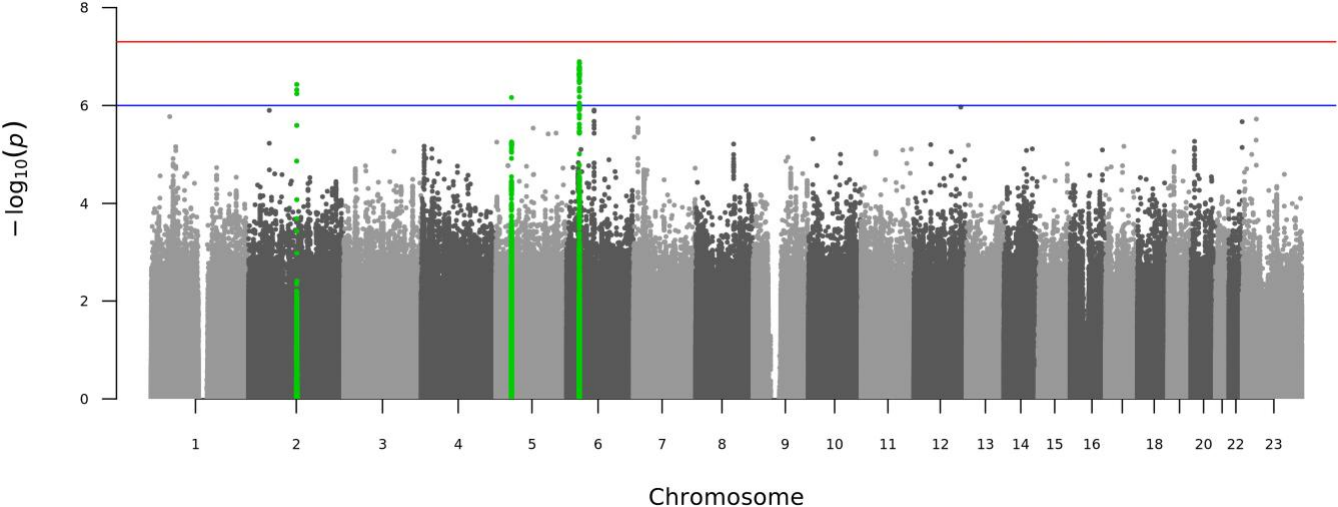
Manhattan plot of the genome-wide association analysis for the risk of upper urinary tract infection for the meta-analysis of both sexes. The x-axis shows the genomic position (chromosomes 1-23; where 23 is the X chromosome), and the y-axis represents the negative logarithm (base 10) of the variant *P* value. The blue line indicates the genome-wide suggestive threshold (*P* ≤ 1E-06), while the red line indicates genome-wide significant threshold (*P* ≤ 5E-08). Genome-wide suggestive loci in the meta-analysis of both sexes (±500 kb of lead variant) are highlighted in green.

In the analysis of both sexes, no SNP reached genome-wide significance, but 3 loci were genome-wide suggestive (Table 3, Figure 2, and Supplementary Figure 3). In addition to the *TSN* locus identified in the female-specific analysis, *LINC00603* on chromosome 5 and *HLA-DQA2* were associated with risk of upper UTIs. Supplementary Figures 4–6 present study-specific Manhattan plots.

**Table 3.** Genetic Variants Associated With Risk of Upper Urinary Tract Infections in the Analysis of Both Sexes

rsid	Chr	Pos^a^	Closest Gene	EA/OA	Meta-analysis						UK Biobank				HUNT				MGI^b^		
					EAF	OR (95% CI)	*P* Value	D	*I^2^*	*I^2^ P* Value	EAF	OR (95% CI)	*P* Value	*INFO*	EAF	OR (95% CI)	*P* Value	*R^2^*	EAF	OR (95% CI)	*P* Value
rs141839823	2	123308687	*TSN*	A/G	0.01	1.97 (1.71–2.24)	3.72E-07	+++	0	8.37E-01	0.01	2.03 (1.65–2.41)	2.76E-04	0.91	0.01	2.11 (1.63–2.58)	2.14E-03	0.81	0.01	1.71 (1.15–2.26)	5.91E-02
rs1113439	5	39907157	*LINC00603*	G/T	0.30	1.13 (1.06–1.18)	6.87E-07	+++	42	1.81E-01	0.30	1.08 (1.00–1.16)	4.50E-02	1	0.31	1.13 (1.04–1.23)	7.23E-03	0.99	0.30	1.21 (1.12–1.30)	4.04E-05
rs3957148	6	32682137	*HLA-DQA2*	G/A	0.11	1.23 (1.15–1.30)	1.27E-07	+++	45	1.62E-01	0.10	1.13 (1.02–1.24)	3.67E-02	1	0.12	1.26 (1.12–1.41)	1.79E-03	0.75	0.11	1.34 (1.20–1.47)	2.96E-05

Only variants with *P* ≤ 1E-06 in the meta-analysis are displayed are displayed.

Abbreviations: Chr, chromosome; CI, confidence interval; D, direction of effect; EA, effect allele; EAF, effect allele frequency; HUNT, Trøndelag Health Study; *I^2^*, heterogeneity; *INFO*, imputation score from IMPUTE2; MGI, Michigan Genomics Initiative; OA, other allele; OR, odds ratio; Pos, chromosome position; *R^2^*, imputation score from Minimac3.

^a^Position of each single-nucleotide polymorphism is given along the chromosome Build 37.

^b^Information about imputation score was not available due to the use of summary-level data.

Quantile-quantile plots did not indicate inflation of the results for any of the conducted analyses (Supplementary Figures 7–12).

### Replication of Top Hits From Previous GWASs on UTIs and Phenome-wide Association Analysis

Six of the 9 previously reported SNPs associated with UTI were available in our study (Supplementary Table 3). No reliable proxies were available for the 3 remaining SNPs. We only replicated 1 association identified by Sakaue et al [6] reported for cystitis (OR, 1.12 [95% CI, 1.08–1.16], *P* = 1.02E-08; effect allele frequency [EAF], 0.26), where we observed an OR of 1.05 (95% CI, 1.00–1.11; *P* = 4.24E-02) and a similar EAF of 0.28.

In The PheWAS analysis, rs3957148 near *HLA-DQA2* and rs11749979 near *SCAMP1- AS1* were strongly associated (*P* ≤ 5E-08) with other traits and diseases (Supplementary Tables 6 and 7). Of note, rs3957148 was associated with an increased risk of traits related to autoimmune diseases, such as type 1 diabetes (OR, 3.94; *P* = 1.00E-310) and rheumatoid arthritis (OR, 3.50; *P* = 1.69E-164). We also found that rs11749979 had a protective effect on traits related to body composition (weight: OR, 0.81; *P* = 7.33E-10).

### Mendelian Randomization Analyses

**Figure 3.**
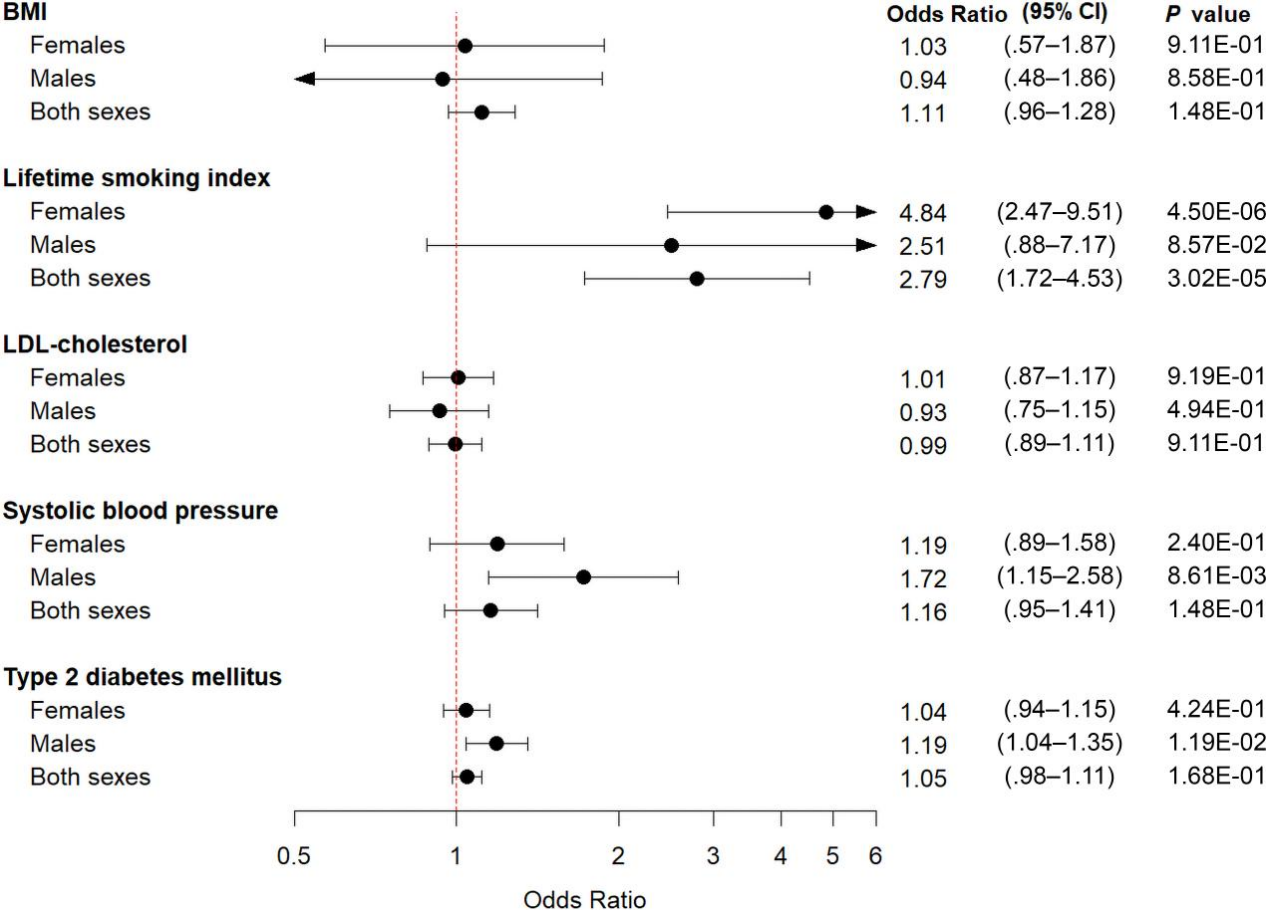
Mendelian randomization analyses of cardiometabolic risk factors on risk of upper urinary tract infections. Forest plot of the 2-sample inverse variance–weighted Mendelian randomization analyses. Each exposure was evaluated separately for the female-only, male-only, and both-sexes analyses. The meta-analysis results from the sex-stratified analysis, which included UK Biobank and the Trøndelag Health Study (HUNT), and the analysis of both sexes, which included UK Biobank, HUNT, and Michigan Genomics Initiative, were used as the outcomes. The x-axis represents the results in odds ratio expressed as per standard deviation increase in genetically proxied levels of the exposure for continuous traits (body mass, smoking, low-density lipoprotein cholesterol, systolic blood pressure) and as per-unit increase in log odds ratio for genetically proxied type 2 diabetes mellitus liability. Abbreviations: BMI, body mass index; CI, confidence interval; LDL, low-density lipoprotein cholesterol.

In the 2-sample MR analysis, smoking was strongly associated with an increased risk of developing upper UTI in the female-only analysis and the analysis of both sexes (Figure 3, Supplementary Figures 13–16, and Supplementary Tables 8 and 9), with ORs of 4.84 (95% CI, 2.47–9.51; *P* = 4.50E-06) and 2.79 (95% CI, 1.72–4.53; *P* = 3.02E-05), respectively. We observed a tendency for a relationship between smoking and the risk of upper UTI among males (OR, 2.51 [95% CI, .88–7.17]; *P* = 8.57E-02). The sensitivity analyses supported this finding, although with very wide CIs. High SBP was found to increase the risk of upper UTIs in the male-only analysis (Figure 3, Supplementary Figures 17 and 18, and Supplementary Table 8), where the OR for developing upper UTIs was 1.72 (95% CI, 1.15–2.58; *P* = 8.61E-03).

We found no strong evidence supporting an association between genetically predicted BMI, LDL-C, and T2DM and the risk of upper UTI in the female-only, male-only, and/or both-sex analyses (Figure 3 and Supplementary Tables 8 and 9), although a nominally significant association was observed for T2DM for the risk of upper UTI in the male-only analysis.

## DISCUSSION

We identified the *TSN* gene to be significantly associated with the risk of upper UTI for females. Four additional loci showed a suggestive association with the risk of upper UTIs among either females, males, or both. Finally, in the MR analyses, we found that smoking was robustly associated with an increased risk of upper UTIs.

The female-only analysis yielded a strong signal near the *TSN* locus, while no effect was observed for the male-only analysis. This may be due to the importance of biological and anatomical differences between females and males. The *TSN* gene encodes a DNA binding protein that recognizes consensus sequences at the breakpoint junctions in chromosomal translocations, involving immunoglobulin/T-cell receptor gene segments [37]. Further research is needed to understand why the identified genetic variants near *TSN* only increased the risk for females. We also observed genetic variants near *DNAI* on chromosome 1 in the female-only analysis, which encodes a member of the dynein intermediate chain family, but its role in upper UTIs is unclear.

In general, there are few GWASs conducted on UTIs, and those have mainly focused on cystitis, self-reported UTIs, or using broad definitions of UTIs, including both cystitis and pyelonephritis [6, 19, 20]. Only 1 previous study has performed female-only and male-only analyses [20]. We replicated 1 association identified by Sakaue et al [6] reported for cystitis among men and women of East Asian ancestry, although the strength of the association was weaker in our study. However, our study did not replicate the association reported for pyelonephritis [6]; despite utilizing a similar phenotype definition, the observed discrepancy in the minor allele frequency of rs17703846, with 23% in European populations (meta-analysis of UK Biobank, HUNT, and MGI) compared to 2% in East Asian populations (BioBank Japan) suggests potential variation in the genetic risk of upper UTI among the included populations.

In the analysis of both sexes, we identified 1 suggestive genetic locus in the human leukocyte antigen (HLA) region, which contains several genes with key roles in the adaptive immune response [38]. The causative allele(s) observed may reside within or near any of the *HLA-DQ* genes in the region due to the extensive LD in this region. The *HLA-DQ* genes all belong to the HLA class II α-chain family and are especially important for bacterial infections since bacteria are processed for presentation by class II major histocompatibility complex (MHC) molecules [39]. *HLA-DQ* has a central role in the peptide loading of the MHC-II molecules, which are expressed by antigen-presenting cells, such as B lymphocytes, dendritic cells, and macrophages, and are recognized by CD4^+^ T cells [40]. Previous studies have found that genetic variants in the HLA region are associated with several infections [19, 20]. To our knowledge, the HLA region has not previously been linked to upper UTIs, but due to its important role in the immune system, it is plausible that SNPs in this region may lead to an increased risk of upper UTIs. Future studies with a larger case population are needed to better understand the role of the HLA region for upper UTIs.

In the analysis of both sexes, we also identified SNPs near *LINC00603* or long intergenic nonprotein coding RNA 603. This gene is a part of the IncRNA class of genes, which does not code for proteins. However, previous studies have indicated that the IncRNA class has important functions in inflammation and immune response [41, 42]. We also observed genetic variants near the secretory carrier membrane protein 1 (*SCAMP1*) and SCAMP1 Antisense RNA 1 (*SCAMP1-AS1*) in the male-only analysis, which is also affiliated with the IncRNA class.

Using 2-sample MR, we identified smoking as a potential risk factor for upper UTIs. While smoking is a well-recognized risk factor for several infections and causes systemic immune dysregulation [26], to our knowledge, this is the first time it has been causally linked to upper UTI using MR analyses. A stronger effect was observed for females compared to the male-only MR analysis, and we also observed a strong effect in the analysis conducted on both sexes. We found limited evidence for the effects of T2DM on the risk of upper UTI, consistent with a previously published MR study [14]. High BMI has previously been found to be a risk factor for UTI [12, 13], but we did not find any evidence of a causal effect of BMI on the risk of upper UTIs. However, this discrepancy may be because the previous studies evaluated a composite of lower and upper UTIs, while ours focused solely on upper UTIs. SBP was found to increase the risk of upper UTI in the male-only analysis, and limited effect was observed for the female-only analysis. We did observe an association between SBP and upper UTI in the MR analysis conducted on both sexes for HUNT. However, this was not replicated in UK Biobank or MGI, and further research is needed to investigate the effect of SBP on upper UTI.

Our study has several strengths and limitations. One major strength is the sex-stratified analysis conducted to explore potential sex differences. Despite using data from multiple large biobanks, we had a relatively small number of cases, which may explain the few genome-wide significant hits. Misclassification of our phenotype might occur due to changes in coding practice, regulation guidelines, and traditions over time and differences in coding practice between different countries. Also, *ICD-9/10* codes serve primarily for billing purposes and are not generated to facilitate research. Individuals used as controls may develop upper UTI after the analyses, which might bias our results in unknown ways. Quality control was performed for each of the 3 datasets to ensure good quality of the included SNPs in the GWAS analysis. However, different genotyping platforms and imputation pipelines between the 3 studies may have impacted our results. As we only evaluated participants of European ancestry, our findings may be less generalizable to other ancestry groups. Ideally, data from separate GWASs should be used for the exposures and outcomes in the 2-sample MR approach to reduce the risk of confounding bias due to overlapping participants [43]. Even though some of the exposures and outcomes were from overlapping cohorts, we observed similar effect estimates when we restricted the analyses to nonoverlapping cohorts, which indicates that sample overlap was not a major issue in this study.

In conclusion, we identified 1 genome-wide significant locus and several loci suggestively associated with an increased risk of upper UTIs. There were clear differences in the genetic risk of upper UTIs between females and males. Further studies using similar phenotype definitions are needed to determine the role of these loci in the etiology of upper UTIs. Through 2-sample MR analyses, we observed that smoking was associated with an increased risk of upper UTI. Knowledge about genetic and modifiable risk factors for upper UTIs is important to improve our understanding of the infection and might enable risk prevention, risk stratification, and individualized therapy in the future. Public health initiatives to reduce smoking may have the additional benefit of reducing the disease burden of upper UTIs.

## Supplementary Data

Supplementary materials are available at *The Journal of Infectious Diseases* online (http://jid.oxfordjournals.org/). Supplementary materials consist of data provided by the author that are published to benefit the reader. The posted materials are not copyedited. The contents of all supplementary data are the sole responsibility of the authors. Questions or messages regarding errors should be addressed to the author.

## Supplementary Material

jiae231_Supplementary_Data

## Notes

***Acknowledgments*.** This project was conducted using data from the UK Biobank resource under application number 40135. The HUNT study is a collaboration between the HUNT Research Centre (Faculty of Medicine and Health Sciences, Norwegian University of Science and Technology), Trøndelag County Council, Central Norway Regional Health Authority, and the Norwegian Institute of Public Health. We want to acknowledge the participants and investigators of the MGI. We used the following web-based resources: Locus Zoom (http://locuszoom.org), Open Targets platform (genetics.opentargets.org), and LDlink (https://ldlink.nih.gov/?tab=home).

***Author contributions.*** All authors contributed substantially to the interpretation of the data and critically revised the manuscript. All authors have approved the submitted version and are personally accountable for their own contributions.

***Data availability.*** Individual-level data from UK Biobank and HUNT are available by application. Summary-level data from MGI are available upon request to MGI. Summary-level statistics from this study will be available in the GWAS catalog at the time of publication.

***Disclaimer.*** The funders had no role in study design, data collection, data analyses, data interpretation, writing of the report, or the decision to submit the article for publication.

***Financial support*.** This work was supported by Liaison Committee between the Central Norway Regional Health Authority and the Norwegian University of Science and Technology.
