# Supplementary material for: A Genome-wide Association Study of Susceptibility to Upper Urinary Tract Infections
Source: J Infect Dis. 2024 May 7;230(6):e1334–43. doi: 10.1093/infdis/jiae231 (PMC11646603; doi:10.1093/infdis/jiae231)
Supplement: jiae231_Supplementary_Data [file jiae231_supplementary_data.zip › Supplemental_material_HeleneMarieFlatby_cleancopy.docx]

**Supplemental material**

**A genome-wide association study of susceptibility to upper urinary tract infections**

Helene M Flatby, PhD^1,2^

Anuradha Ravi, PhD^1,3^

Kristin V Liyanarachi, MD^1,4^

Jan E Afset, MD PhD^1,5,6^

Humaira Rasheed, PhD^7,8^

Ben M Brumpton, MPH PhD^7,8,9^

Kristian Hveem, MD PhD^7,10^

Bjørn O Åsvold, MD PhD^11,9, 7^

Andrew T DeWan, MPH PhD^12,1^

Erik Solligård, MD PhD^1,13^

Jan K Damås, MD PhD^1,14,4^

Tormod Rogne, MD PhD^12,1^

1. Mid-Norway Centre for Sepsis Research, Department of Circulation and Medical Imaging, NTNU, Norwegian University of Science and Technology, Trondheim, Norway
2. Clinic of Anaesthesia and Intensive Care, St. Olavs Hospital, Trondheim University Hospital, Trondheim, Norway
3. Department of Medical Genetics, St. Olavs Hospital, Trondheim University Hospital, Trondheim, Norway
4. Department of Infectious Diseases, St. Olavs Hospital, Trondheim University Hospital, Trondheim, Norway
5. Department of Clinical and Molecular Medicine, Norwegian University of Science and Technology, Trondheim, Norway
6. Department of Medical Microbiology, St. Olavs Hospital, Trondheim University Hospital, Trondheim, Norway
7. K.G. Jebsen Center for Genetic Epidemiology, Department of Public Health and Nursing, NTNU, Norwegian University of Science and Technology, Trondheim, Norway
8. Clinic of Medicine, St. Olavs Hospital, Trondheim University Hospital, Trondheim, Norway
9. HUNT Research Center, Department of Public Health and Nursing, NTNU, Norwegian University of Science and Technology, Levanger, Norway
10. Department of Research, Innovation, and Education, St. Olavs Hospital, Trondheim University Hospital, Trondheim, Norway
11. Department of Endocrinology, Clinic of Medicine, St. Olavs Hospital, Trondheim University Hospital, Trondheim, Norway
12. Department of Chronic Disease Epidemiology and Center for Perinatal, Pediatric and Environmental Epidemiology, Yale School of Public Health, New Haven, CT, USA
13. Helse Møre og Romsdal Hospital Trust, Ålesund, Norway
14. Centre of Molecular Inflammation Research, Department of Clinical and Molecular Medicine, NTNU, Norwegian University of Science and Technology, Trondheim, Norway

**Corresponding author:**

Helene Marie Flatby

Department of Circulation and Medical Imaging, NTNU

Prinsesse Kristinas gate 3, Akutten og Hjerte-lunge-senteret, 3. etg

Trondheim 7491, Norway

Email: helene.flatby@ntnu.no

Phone: +47 472 65 442

ORCiD: <https://orcid.org/0000-0002-5700-020X>

**Keywords:** Upper urinary tract infection; Pyelonephritis; genome-wide association study; Mendelian randomization; human leukocyte antigen; smoking

**Short title:** Risk of upper urinary tract infection

**SUPPLEMENTARY TEXT**

**Cohort’s description.**

*UK Biobank*

The protocol for UK Biobank with information about recruitment, design and sample characteristics has been described in detail elsewhere, including information about genotyping, quality control, and imputation [1, 2]. In short, UK Biobank is a longitudinal community-based study in the United Kingdom conducted from 2006 to 2010, where 9.2 million individuals were invited, and 5.5% (503,325) individuals participated. Of the recruited individuals, 407,888 had data from electronic medical records, survey data on lifestyle indicators, and genotype information and were of European ancestry [1, 2].

*The HUNT Study (HUNT)*

The HUNT Study (HUNT; The Trøndelag Health Study, or "Helseundersøkelsen i Trøndelag" [Norwegian]) protocol has also been described in detail elsewhere, including information about genotyping, quality control, and imputation [3]. In brief, HUNT is a population-based cohort where all inhabitants in Nord-Trøndelag county aged 20 years and older were invited to participate [4]. For this study, we used participants from the HUNT2 (1995–1997) and HUNT3 (2006–2008) surveys, where 69.5% (65,236) [5] and 54.1% (50,807) of invitees participated, respectively [4]. Of the recruited individuals, 69,420 had data from electronic medical records, survey data on lifestyle indicators, and genotype information and were of European ancestry.

*Michigan Genomics Initiative (MGI)*

We used summary-level data from the Michigan Genomics Initiative (MGI). The study protocol is described elsewhere, including information regarding genotyping, quality control, and imputation [6]. In short, participants were primarily recruited while awaiting a diagnostic or interventional procedure, either at the preoperative appointment or on the day of their operative procedure at Michigan Medicine. Of the recruited individuals, 39,173 had data from electronic medical records and genotype information and were of European ancestry [6].

**Genotyping**

*UK biobank*

The genotyping was performed using either Affymetrix UK BiLEVE Axiom or the Affymetrix UK Biobank Axiom® array [1]. The genotyped variants were further pre-phased using SHAPEIT3 [7] and imputed utilizing a combination of the UK10K [8], Haplotype Reference Consortium [8], and 1000 Genomes Phase 3 [9] reference panels by IMPUTE4 software [10]. Variants with imputation r^2^ < 0.3 were excluded from further analysis.

*HUNT*

The adult participants in HUNT2 and HUNT3 were genotyped using one of three different Illumina HumanCoreExome arrays (HumanCoreExome12 v1.0, HumanCoreExome12 v1.1, and UM HUNT Biobank v1.0), as described previously [11].

The quality control of all genotyped data was performed, where variants meeting any of the following criteria were excluded: call rate < 99%, large chromosomal copy number variants, contamination > 2.5% as estimated with BAF Regress [12], genotypic and phenotypic sex discordance, and not of European ancestry, the *P* value for departure from Hardy-Weinberg equilibrium (*P* value < 0.0001). We conducted whole-genome imputation with Minimac4 (v1.4.1), using the customized Haplotype Reference consortium release 1.1 (HRC v1.1 2016) [13] for autosomal variants and HRC v1.1 for chromosome X variants. Variants with imputation r^2^ < 0.3 were excluded from further analysis.

*MGI*

The genotyping was performed using customized Illumina Infinium CoreExome-24 array [6]. The genotyped variants were further pre-phased using EAGLE (v2.4.1)[14] without the use of a reference panel (“within-cohort” phasing). The imputation was performed by using both the Haplotype Reference Consortium (HRC) reference panel [15] and the Trans-Omics for Precision Medicine (TOPMed) reference panel [16] by IMPUTE4 (v1.0.0) software [10]. Variants with imputation r^2^ < 0.3 were excluded from further analysis [6].

**Genome-wide association analyses**

*UK Biobank*

In the genome-wide association analyses, we used a generalized linear mixed model with the saddle point approximation implemented in SAIGE (version 0.35.8.3) to account for any residual relatedness structure and for case-control imbalance [17]. We used the imputed dosages for the analysis, where genetic variants were on NCBI build 37 (hg19). For chromosome 23, males were coded as if they were homozygous diploid for the observed allele. The same covariates were included in the analyses of the autosomal chromosomes and for chromosome 23, where we adjusted for birth year, sex, genotype chip, and ancestry-informative principal components 1 to 6. Exclusions were made for variants with a minor allele frequency (MAF) of < 0.5% and an imputation score < 0.3, resulting in 11,759,910 variants.

*HUNT*

We performed the GWAS using SAIGE (version 0.35.8.8) [17] for the autosomal chromosomes, while BOLT-LMM (version 2.3.4) [18] was used in the analyses of chromosome 23, coding males as homozygous diploid for the observed allele. The beta-coefficients from BOLT-LMM were transformed using the following formula: log odds ratio (OR) = β / (μ * (1 - μ)), where β = the single-nucleotide polymorphism (SNP) effect estimate on the quantitative scale, and μ = case fraction [14]. The standard errors from BOLT-LMM were transformed by: SE_transformed_ = SE_original_ / (μ * (1 - μ)) [14]. As for the GWAS conducted for UK Biobank, we used the imputed dosages for the analysis, and genetic variants were on NCBI build 37 (hg19). The GWASs were adjusted for birth year, sex, genotype chip, and ancestry-informative principal components 1 to 5. The same SNP-exclusions used for UK Biobank were also applied for HUNT, where variants with MAF < 0.5% and imputation score < 0.3 were excluded, resulting in 9,108,680 variants.

*MGI*

GWAS analysis was conducted by the MGI using SAIGE (version 0.43.3) [17], adjusting for genetic relatedness, age, sex, chip version, and the first 4 principal components. The same SNP exclusions used for the other two datasets were applied for MGI, where variants with MAF < 0.5% and imputation score < 0.3 were excluded. Prior to the meta-analysis, the MGI summary results were remapped from NCBI build 38 (hg38) to NCBI build 37 (hg19) using the LiftOver tool [19] to align with the other two studies, resulting in 10,556,728 variants after the remapping.

*Identification of statistically significant and independent variants*

Linkage disequilibrium (LD)-independent genetic signals were identified by clumping (*r^2^* = 0.2 and 500 kb window) using PLINK (version 1.9) and 1000 Genomes EUR population Phase 3 [9] as the reference panel for estimating LD. We considered associations with *P* ≤ 5E-08 to be genome-wide significant and associations with *P* ≤ 1E-06 to be genome-wide suggestive.

**Exposure GWAS cohorts**

Genetic variants to be used as instruments for the exposures were obtained from publicly available GWAS summary data sources. Independent genetic variants were identified using clumping (*r^2^* < 0.001 within 10,000 kb windows, and *P* ≤ 5E-08), while the variance explained was estimated using get_r_from_pn(), as implemented in the TwoSample MR package[20]. A brief description of each exposure GWAS is described below.

**BMI**

The genetic instruments used for BMI were extracted from a meta-analysis conducted on both sexes using 681,275 individuals of European ancestry from the GIANT consortium, which includes UK Biobank [21]. Through the GIANT consortium website (<https://portals.broadinstitute.org/collaboration/giant/index.php/GIANT_consortium_data_files>), we were able to retrieve female-only and male-only analysis data [22]. BMI was measured or self-reported in kg per height in meters squared. The analysis was adjusted for age, age squared, and any necessary study-specific covariates, such as principal components. For the analysis of both sexes, 595 independent genetic loci were identified after clumping, which explained 5.4% of the variance. The female-only analysis consisted of approximately 67,670 individuals, where 11 independent genetic loci were identified, and the variance explained was 0.87%. The male-only analysis consisted of approximately 58,588 individuals, where 10 independent genetic loci were identified, and the variance explained was 0.88%.

**Lifetime smoking**

The genetic instruments used for lifetime smoking were extracted from a GWAS conducted using 462,690 individuals of European ancestry from UK Biobank, where 249,318 were never smokers (54%), 164,649 were former smokers (36%), and 48,723 were current smokers (11%). The GWAS was conducted to capture lifetime smoking by using smoking heaviness, duration, and imitation. 124 independent genome-wide significant variants were identified after clumping. The mean value of lifetime smoking score was 0.36 (SD =0.69) [23], and the variance explained was 1.19%. Since no female-only and/or male-only GWAS for lifetime smoking were available, the analysis conducted on both sexes was used as the exposure for all MR analyses.

**Low-density lipoprotein cholesterol**

The genetic instruments used for low-density lipoprotein cholesterol (LDL-C) were extracted from a meta-analysis using 188,577 individuals from a meta-analysis of 37 studies of European ancestry. Blood lipid levels were measured after >8 hours of fasting, and individuals on lipid-lowering medications were excluded when possible. The analysis was adjusted for age, sex, principal components, and genomic control [24]. 78 independent genetic loci were identified after clumping, which explained 8.73% of the variance. Since no female-only and/or male-only GWAS for LDL-C were available, the analysis conducted on both sexes was used as the exposure for all MR analyses.

**Systolic blood pressure**

The genetic instruments used for systolic blood pressure were extracted from a GWAS using 318,417 individuals of European ancestry from UK Biobank. Blood pressure was recorded at the baseline assessment Centre for all participants, where each reading was taken twice, two minutes apart. The GWAS analysis was performed to obtain genetic variants used as instruments for a two-sample MR analysis. In the GWAS analysis, the second reading and missing data were replaced with the first measure, or any follow-up assessment Centre measures. The GWAS analysis was adjusted for sex, age, and principal components [25]. 189 independent genetic loci were identified after clumping, which explained 3.2% of the variance. Since no female-only and/or male-only GWAS for systolic blood pressure were available, the analysis conducted on both sexes was used as the exposure for all MR analyses.

**Type 2 diabetes mellitus**

The genetic instruments used for type 2 diabetes mellitus were extracted from an analysis conducted on both sexes using 898,130 (74,124 cases and 824,006) individuals of European ancestry from the DIAMANTE consortium, which combines data from 32 GWASs, including GWAS results from UK Biobank and MGI [26]. Through the DIAMANTE consortium (<https://diagram-consortium.org/downloads.html>), we were able to retrieve female-only and male-only analysis data [26]. For the analysis conducted on both sexes, 99 independent genetic loci were identified after clumping, which explained 5.72% of the variance. The female-only analysis consisted of approximately 96,258 individuals, where 74 independent genetic loci were identified, and the variance explained was 4.82%. The male-only analysis consisted of approximately 127,759 individuals, where 99 independent genetic loci were identified, and the variance explained was 5.72%.

**SUPPLEMENTARY TABLES**

| **Supplementary Table 1.** Field ID used in UK Biobank. | | | |
| --- | --- | --- | --- |
| **Used for** | **Field ID** | **Description** | **Category** |
| GWAS analyses | 22001 | Genetic sex | Genotyping process and sample QC |
|  | 31 | Sex | Baseline characteristics |
|  | 22006 | Genetic ethnic grouping | Genotyping process and sample QC |
|  | 22009 | Genetic principal components | Genotyping process and sample QC |
|  | 34 | Year of birth | Genotyping process and sample QC |
|  | 22000 | Genotype measurement batch | Genotyping process and sample QC |
|  | 41270 | Diagnoses - ICD10 | Summary Diagnoses |
|  | 41271 | Diagnoses - ICD9 | Summary Diagnoses |
| Background characteristics table | 20160 | Ever smoked | Smoking |
|  | 21001 | Body mass index (BMI) | Body size measures |
|  | 23526 | Cholesterol in IDL | NMR metabolomics |
|  | 2443 | Diabetes diagnosed by doctor | Medical conditions |
|  | 30690 | Cholesterol | Blood biochemistry |
|  | 4080 | Systolic blood pressure, automated reading | Blood pressure |
|  | 21022 | Age at recruitment | Baseline characteristics |

| **Supplementary Table 2.** Phenotype definition in previous GWASs on urinary tract infections. | | | |
| --- | --- | --- | --- |
| **Study** | **Phenotype name** | **Cohorts** | **Phenotype definition** |
| Tängdén et al. [27] | UTI | UK Biobank | ICD-10 codes: N30.0, N30.9, N10, N15.1, N39.0, N41.0, N41.3 |
|  | Cystitis | UK Biobank | ICD-10 codes: N30.0, N30.9 |
| Tian et al. [28] | UTI frequency | 23andMe | Self-reported UTI based on questionnaire data |
| Sakaue et al. Sakaue et al. [29] | Pyelonephritis | BioBank Japan | ICD-10 code: N10 |
|  |  | UK Biobank | PheCode^a^: 590  ICD-10 codes: N10, N11, N11.0, N11.1, N11.8, N11.9, N12, N13.6, N15.1, N15.9, N16 |
|  |  | FinnGen R3 | FinnGen R3 endpoint^b^: N14_PYELONEPHR  ICD-10 code: N10 |
|  | Cystitis | BioBank Japan | ICD-10 code: N30.0 |
|  |  | UK Biobank | PheCode^a^: 592.11  ICD-10 code: N30.0 |
|  |  | FinnGen R3 | FinnGen R3 endpoint^b^: N14_CYSTITIS  ICD-10 code: N30.0 |
| ^a^ PheCode for UK Biobank available at https://phewascatalog.org/phecodes_icd10  ^b^ FinnGen R3 endpoint available at https://r3.risteys.finngen.fi/ | | | |

| **Supplementary Table 3.** Comparison with previous GWASs on urinary tract infections. | | | | | | | | | | | | | | | | |
| --- | --- | --- | --- | --- | --- | --- | --- | --- | --- | --- | --- | --- | --- | --- | --- | --- |
|  | | | | | | | | | | | | |  | **Upper UTI meta-analysis^b^** | | |
| **Study** | **Cohort** | **Phenotype** | **Cases** | **Controls** | **rsid** | **CHR** | **Pos^c^** | **Closest gene** | **EA/OA** | **EAF^a^** | **OR/effect**  **(95% CI)** | ***P*** |  | **EAF** | **OR**  **(95% CI)** | ***P*** |
| Tängdén et al. [27] | UK Biobank | UTI | 13,754 | 323,730 | rs771331833 | 8 | 118513358 | *MED30* | A/C | - | 2.99  (2.10 - 4.26) | 1.26E-09 |  | - | - | - |
|  |  |  |  |  | - | 3 | 47875364 | *DHX30* | AT/A | - | 1.47  (1.29 - 1.68) | 2.81E-08 |  | 0.01 | 1.05  (0.69 - 1.42) | 0.77 |
|  |  |  |  |  | rs189319388 | 3 | 7867797 | *GRM7* | C/T | - | 1.53  (1.31-1.78) | 4.63E-08 |  | 0.01 | 1.28  (0.76- 1.28)^d^ | 0.35 |
|  |  | Cystitis | 1,457 | 336,027 | rs192629083 | 7 | 14037179 | *ETV1* | T/C | - | 3.70  (2.40 - 5.72) | 3.50E-09 |  | - | - | - |
|  |  |  |  |  | rs77261774 | 4 | 60641124 | *LINC02429* | A/G | - | 1.79  (1.46 - 2.19) | 1.49E-08 |  | 0.02 | 1.01  (0.85 - 1.18) | 0.86 |
| Tian et al. [28] | 23andMe | UTI frequency | 35,000 | 33,478 | rs2976388 | 8 | 143760256 | *PSCA* | G/A | 0.56 | 0.04  (0.04 - 0.05) ^g^ | 3.27E-10 |  | 0.58 | 1.03  (0.98-1.07) | 0.25 |
|  |  |  |  |  | rs146906133 | 15 | 44262951 | *FRMD5* | T/C | 1.00 | 0.38  (0.32 - 0.45) ^g^ | 2.02E-08 |  | - | - | - |
| [29] | BioBank Japan | Pyelonephritis | 1,811 | 175,288 | rs17703846 | 14 | 45205943 | *LINC02302* | T/C | 0.02 | 2.30  (2.01 - 2.60) | 2.41E-08 |  | 0.23 | 1.00  (0.95 - 1.06) | 0.96 |
|  | BioBank Japan,  UK Biobank, and FinnGen | Cystitis | 6,586 | 625,982 | rs12154203 | 6 | 100283110 | *MCHR2* | C/A | 0.26 | 1.12  (1.08 - 1.16) | 1.02E-08 |  | 0.28 | 1.05  (1.00 - 1.11) | 4.24E-02 |
| Missing data denoted -.  ^a^ Only results with MAF > 0.5% are presented.  ^b^ Meta-analysis of UK Biobank, HUNT and MGI for both sexes.  ^c^ Position of each SNP is given along the chromosome Build 37.  ^d^ Only found in UK Biobank.  ^g^ Effect estimate (not OR) for quantitative trait.  Abbreviations: CHR, chromosome; CI, confidence interval; EA, effect allele; EAF, effect allele frequency; OA, other allele; OR, odds ratio; Pos, chromosome position; UTI, urinary tract infection. | | | | | | | | | | | | | | | | |

| **Supplementary Table 8.** Mendelian randomization sensitivity analyses of cardiometabolic risk factors on the risk of urinary tract infections for the female-only and male-only analysis. | | | | | | | | | |
| --- | --- | --- | --- | --- | --- | --- | --- | --- | --- |
|  |  | **Female-only** | | | | **Male-only** | | | |
| **Exposure^a^** | **Methode** | **N** | **OR^b^**  **(95% CI)** | ***P* value** | **Cochran's**  **Q**  ***P* value** | **N** | **OR^b^**  **(95% CI)** | ***P* value** | **Cochran's**  **Q**  ***P* value** |
| BMI | MR Egger | 11 | 1.16  (0.21 - 6.48) | 8.66E-01 | 7.47E-01 | 10 | 8.01  (0.72 - 89.40) | 1.29E-01 | 6.07E-01 |
|  | Weighted  median | 11 | 0.99  (0.55-1.77) | 9.65E-01 |  | 10 | 1.46  (0.60 - 3.54) | 4.04E-01 |  |
|  | IVW | 11 | 1.28  (0.83-1.98) | 2.59E-01 | 8.20E-01 | 10 | 0.94  (0.48 - 1.86) | 8.58E-01 | 3.81E-01 |
|  | Simple mode | 11 | 0.99  (0.40 - 2.46) | 9.78E-01 |  | 10 | 0.86  (0.16- 4.61) | 8.65E-01 |  |
|  | Weighted  mode | 11 | 0.94  (0.46- 1.98) | 8.75E-01 |  | 10 | 1.60  (0.61- 4.21) | 3.69E-01 |  |
|  | Egger  intercept |  | 1.01  (0.92 - 4.33) | 9.10E-01 |  |  | 0.89  (0.76 - 1.02) | 1.08E-01 |  |
| Smoking | MR Egger | 118 | 4.05  (0.28 - 58.28) | 3.05E-01 | 2.80E-01 | 118 | 70.35  (1.71 - 4239.11) | 4.43E-02 | 8.98E-02 |
|  | Weighted  median | 118 | 6.74  (2.56 - 17.80) | 1.16E-04 |  | 118 | 4.92  (1.15 - 21.10) | 3.19E-02 |  |
|  | IVW | 118 | 4.84  (2.47 - 9.51) | 4.50E-06 | 3.02E-01 | 118 | 2.51  (0.88 - 7.17) | 8.57E-02 | 7.13E-02 |
|  | Simple mode | 118 | 19.96  (1.23 - 325.24.99) | 3.76E-02 |  | 118 | 8.11  (0.18 - 370.06) | 2.85E-01 |  |
|  | Weighted  mode | 118 | 18.86  (1.68 - 211.72) | 1.89E-02 |  | 118 | 8.11  (0.27 - 247.73) | 2.33E-01 |  |
|  | Egger  intercept |  | 1.00  (0.97 - 1.03) | 8.93E-01 |  |  | 0.96  (0.92 - 1.01) | 1.02E-01 |  |
| LDL-C | MR Egger | 76 | 0.95  (0.76 - 1.18) | 6.17E-01 | 3.07E-01 | 76 | 0.96  (0.71 - 1.31) | 8.08E-01 | 6.65E-01 |
|  | Weighted  median | 76 | 1.04  (0.82 - 1.31) | 7.48E-01 |  | 76 | 0.94  (0.68 - 1.30) | 7.10E-01 |  |
|  | IVW | 76 | 1.01  (0.87 - 1.17) | 9.19E-01 | 3.17E-01 | 76 | 0.93  (0.75 - 1.15) | 4.94E-01 | 6.91E-01 |
|  | Simple mode | 76 | 1.13  (0.76 - 1.68) | 5.39E-01 |  | 76 | 1.01  (0.56 - 1.84) | 9.67E-01 |  |
|  | Weighted  mode | 76 | 1.01  (0.83 - 1.23) | 9.18E-01 |  | 76 | 0.92  (0.69 - 1.24) | 5.95E-01 |  |
|  | Egger  intercept |  | 1.01  (0.99 - 1.02) | 4.31E-01 |  |  | 1.00  (0.98 - 1.02) | 7.53E-01 |  |
| SBP | MR Egger | 186 | 1.11  (0.45 - 2.73) | 8.18E-01 | 2.41E-01 | 186 | 1.75  (0.49 - 6.20) | 3.89E-01 | 8.08E-01 |
|  | Weighted  median | 186 | 1.13  (0.73 - 1.73) | 5.87E-01 |  | 186 | 1.11  (0.60 - 2.04) | 7.37E-01 |  |
|  | IVW | 186 | 1.19  (0.89 - 1.58) | 2.40E-01 | 2.57E-01 | 186 | 1.72  (1.15 - 2.58) | 8.61E-03 | 8.22E-01 |
|  | Simple mode | 186 | 1.06  (0.29 - 3.78) | 9.33E-01 |  | 186 | 1.11  (0.23 - 5.38) | 8.94E-01 |  |
|  | Weighted  mode | 186 | 0.99  (0.34 - 2.93) | 9.91E-01 |  | 186 | 1.02  (0.27 - 3.87) | 9.79E-01 |  |
|  | Egger  intercept |  | 1.00  (0.97 - 1.03) | 8.79E-01 |  |  | 1.00  (0.97 - 1.03) | 9.79E-01 |  |
| *Continued on next page.* | | | | | | | | | |
|  |  |  |  |  |  |  |  |  |  |
|  |  |  |  |  |  |  |  |  |  |
|  |  |  |  |  |  |  |  |  |  |
|  |  |  |  |  |  |  |  |  |  |
|  |  |  |  |  |  |  |  |  |  |
|  |  |  |  |  |  |  |  |  |  |
| **Supplementary Table 8.** *Continued.* | | | | | | | | | |
| T2DM | MR Egger | 72 | 0.91  (0.71 - 1.17) | 4.74E-01 | 8.86E-01 | 96 | 1.37  (1.02 - 1.84) | 4.22E-02 | 8.89E-01 |
|  | Weighted  median | 72 | 0.93  (0.79 - 1.10) | 4.17E-01 |  | 96 | 1.19  (0.97 - 1.45) | 9.70E-02 |  |
|  | IVW | 72 | 1.04  (0.94 - 1.15) | 4.24E-01 | 8.78E-01 | 96 | 1.19  (1.04 - 1.35) | 1.19E-02 | 8.86E-01 |
|  | Simple mode | 72 | 0.91  (0.65 - 1.29) | 6.00E-01 |  | 96 | 0.96  (0.59 - 1.55) | 8.65E-01 |  |
|  | Weighted  mode | 72 | 0.93  (0.76 - 1.13) | 4.64E-01 |  | 96 | 1.26  (0.96 - 1.67) | 1.04E-01 |  |
|  | Egger  intercept |  | 1.00  (0.99 - 1.01) | 4.76E-01 |  |  | 1.01  (0.99 - 1.04) | 2.97E-01 |  |
| ^a^ Only independent SNPs (*r^2^* < 0.001 within 10,000 kb windows), strongly associated (*P* ≤ 5E-08) were used as genetic instruments for the exposure.  ^b^ The odds ratios (OR) correspond to a 1 standard deviation increase for continuous traits (BMI, smoking, LDL-C, and SBP) and as per unit increase in the log odds ratio for genetically proxied T2DM liability.  Abbreviations: BMI, body mass index; CI, confidence interval; IVW, inverse-variance weighted; LDL-C, low-density lipoprotein cholesterol; Michigan Genomics Initiative, MGI; N, number of SNPs used; SBP, systolic blood pressure; Smoking, Lifetime smoking index; T2DM, type 2 diabetes mellitus. | | | | | | | | | |

| **Supplementary Table 9.** Mendelian randomization sensitivity analyses of cardiometabolic risk factors on the risk of urinary tract infections for the analysis of both sexes. | | | | | | | | | | | | | | | | | |
| --- | --- | --- | --- | --- | --- | --- | --- | --- | --- | --- | --- | --- | --- | --- | --- | --- | --- |
|  |  | **Meta-Analysis** | | | | **UK Biobank** | | | | **HUNT** | | | | **MGI** | | | |
| **Exposure^a^** | **Methode** | **N** | **OR^b^**  **(95% CI)** | ***P* value** | **Cochran's**  **Q**  ***P* value** | **N** | **OR^b^**  **(95% CI)** | ***P* value** | **Cochran's**  **Q**  ***P* value** | **N** | **OR^b^ (95% CI)** | ***P* value** | **Cochran's**  **Q**  ***P* value** | **N** | **OR^b^**  **(95% CI)** | ***P* value** | **Cochran's**  **Q**  ***P* value** |
| BMI | MR Egger | 511 | 0.94  (0.65 - 1.35) | 7.21E-01 | 1.47E-01 | 510 | 0.70  (0.39 - 1.23) | 2.10E-01 | 6.91E-02 | 510 | 1.06  (0.55 - 2.07) | 8.58E-01 | 9.14E-01 | 508 | 1.25  (0.65 - 2.43) | 5.02E-01 | 3.59E-01 |
|  | Weighted  median | 511 | 1.04  (0.81 - 1.34) | 7.46E-01 |  | 510 | 0.96  (0.66 - 1.39) | 8.20E-01 |  | 510 | 0.88  (0.56 - 1.40) | 6.04E-01 |  | 508 | 1.25  (0.80 - 1.94) | 3.31E-01 |  |
|  | IVW | 511 | 1.11  (0.96 - 1.28) | 1.48E-01 | 1.47E-01 | 510 | 1.04  (0.83 - 1.30) | 7.47E-01 | 6.37E-02 | 510 | 1.06  (0.82 - 1.37) | 6.66E-01 | 9.19E-01 | 508 | 1.27  (0.98 - 1.65) | 6.66E-02 | 3.71E-01 |
|  | Simple mode | 511 | 1.16  (0.55 - 2.44) | 6.90E-01 |  | 510 | 0.81  (0.25 - 2.60) | 7.21E-01 |  | 510 | 0.72  (0.20 - 2.64) | 6.24E-01 |  | 508 | 0.97  (0.23 - 4.17) | 9.70E-01 |  |
|  | Weighted  mode | 511 | 1.09  (0.70 - 1.71) | 6.98E-01 |  | 510 | 0.62  (0.32 - 1.22) | 1.70E-01 |  | 510 | 0.72  (0.33 - 1.58) | 4.18E-01 |  | 508 | 1.13  (0.49 - 2.6) | 7.66E-01 |  |
|  | Egger  intercept |  | 1.00  (1.00 - 1.01) | 3.17E-01 |  |  | 1.01  (1.00 - 1.02) | 1.34E-01 |  |  | 1.00  (0.99 - 1.01) | 9.91E-01 |  |  | 1.00  (0.99 - 1.01) | 9.60E-01 |  |
| Smoking | MR Egger | 118 | 3.99  (0.59 - 26.93) | 1.58E-01 | 1.51E-01 | 118 | 12.27  (0.67 - 124.44) | 9.35E-02 | 1.57E-01 | 117 | 7.57  (0.22 - 162.88) | 2.66E-01 | 2.43E-01 | 118 | 0.41  (0.01 - 11.19) | 5.95E-01 | 7.15E-01 |
|  | Weighted  median | 118 | 2.47  (1.25 - 4.88) | 8.92E-03 |  | 118 | 2.18  (0.77 - 6.18) | 1.43E-01 |  | 117 | 5.40  (1.50 - 19.45) | 9.83E-03 |  | 118 | 1.4  (0.40 - 4.94) | 5.99E-01 |  |
|  | IVW | 118 | 2.79  (1.72 - 4.53) | 3.02E-05 | 1.64E-01 | 118 | 3.19  (1.52 - 6.69) | 2.23E-03 | 1.58E-01 | 117 | 4.78  (1.96 - 11.64) | 5.74E-04 | 2.62E-01 | 118 | 1.39  (0.60 - 3.22) | 4.43E-01 | 7.24E-01 |
|  | Simple  mode | 118 | 3.32  (0.52 - 20.99) | 2.05E-01 |  | 118 | 0.91  (0.05 - 15.97) | 9.46E-01 |  | 117 | 7.97  (0.27 - 160.70) | 2.32E-01 |  | 118 | 1.97  (0.08 - 48.19) | 6.80E-01 |  |
|  | Weighted  mode | 118 | 3.07  (0.63 - 15.02) | 1.69E-01 |  | 118 | 1.03  (0.06 - 18.09) | 9.84E-01 |  | 117 | 5.56  (0.31 - 101.21) | 2.49E-01 |  | 118 | 1.6  (0.07 - 35.01) | 7.67E-01 |  |
|  | Egger  intercept |  | 1.01  (0.99 - 1.03) | 7.06E-01 |  |  | 1.02  (0.99 - 1.05) | 3.49E-01 |  |  | 1.02  (0.98 - 1.06) | 7.93E-01 |  |  | 1.02  (0.98 - 1.05) | 4.54E-01 |  |
| LDL-C | MR Egger | 76 | 0.95  (0.81 - 1.13) | 5.85E-01 | 4.34E-02 | 76 | 0.89  (0.71 - 1.13) | 3.43E-01 | 2.44E-01 | 76 | 1.05  (0.79 - 1.39) | 7.30E-01 | 4.30E-01 | 76 | 0.96  (0.74 - 1.26) | 7.89E-01 | 5.41E-01 |
|  | Weighted  median | 76 | 1.07  (0.90 - 1.28) | 4.26E-01 |  | 76 | 1.05  (0.82 - 1.34) | 7.04E-01 |  | 76 | 0.97  (0.73 - 1.29) | 8.11E-01 |  | 76 | 1.12  (0.82 - 1.52) | 4.89E-01 |  |
|  | IVW | 76 | 0.99  (0.89 - 1.11) | 9.11E-01 | 4.73E-02 | 76 | 0.95  (0.81 - 1.12) | 5.60E-01 | 2.53E-01 | 76 | 1.02  (0.85 - 1.24) | 8.12E-01 | 4.60E-01 | 76 | 1.03  (0.85 - 1.23) | 7.91E-01 | 5.61E-01 |
|  | Simple  mode | 76 | 1.00  (0.73 - 1.38) | 9.77E-01 |  | 76 | 0.82  (0.53 - 1.27) | 3.73E-01 |  | 76 | 1.11  (0.65 - 1.91) | 7.06E-01 |  | 76 | 0.97  (0.56 - 1.70) | 9.16E-01 |  |
|  | Weighted  mode | 76 | 1.02  (0.88 - 1.17) | 8.23E-01 |  | 76 | 0.98  (0.78 - 1.24) | 8.92E-01 |  | 76 | 0.96  (0.75 - 1.23) | 7.59E-01 |  | 76 | 1.06  (0.79 - 1.43) | 6.97E-01 |  |
|  | Egger  intercept |  | 1.00  (0.99 - 1.01) | 5.16E-01 |  |  | 1.01  (0.99 - 1.02) | 4.46E-01 |  |  | 1.00  (0.98 - 1.02) | 8.02E-01 |  |  | 1.01  (0.99 - 1.02) | 5.31E-01 |  |
| *Continued on next page* | | | | | | | | | | | | | | | | |  |
| **Supplementary Table 9.** *Continued* | | | | | | | | | | | | | | | | | |
| SBP | MR Egger | 185 | 1.32  (0.71 - 2.46) | 3.80E-01 | 3.05E-01 | 186 | 0.69  (0.27 - 1.75) | 4.36E-01 | 4.07E-01 | 184 | 3.35  (1.08 - 10.42) | 3.82E-02 | 8.25E-01 | 184 | 1.4  (0.44 - 4.47) | 5.70E-01 | 2.86E-01 |
|  | Weighted  median | 185 | 1.14  (0.85 - 1.51) | 3.84E-01 |  | 186 | 0.94  (0.61 - 1.47) | 7.97E-01 |  | 184 | 2.10  (1.23 - 3.58) | 6.45E-03 |  | 184 | 1.18  (0.69 - 2.04) | 5.48E-01 |  |
|  | IVW | 185 | 1.16  (0.95 - 1.41) | 1.48E-01 | 3.20E-01 | 186 | 1.02  (0.76 - 1.38) | 8.94E-01 | 4.11E-01 | 184 | 2.02  (1.41 - 2.91) | 1.43E-04 | 8.27E-01 | 184 | 0.81  (0.56 - 1.16) | 2.49E-01 |  |
|  | Simple  mode | 185 | 1.32  (0.56 - 3.09) | 5.29E-01 |  | 186 | 0.91  (0.22 - 3.77) | 8.94E-01 |  | 184 | 1.55  (0.33 - 7.26) | 5.78E-01 |  | 184 | 1.2  (0.24 - 5.98) | 8.24E-01 | 2.86E-01 |
|  | Weighted  mode | 185 | 1.17  (0.56 - 2.44) | 6.78E-01 |  | 186 | 0.80  (0.24 - 2.65) | 7.16E-01 |  | 184 | 2.12  (0.68 - 6.61) | 1.99E-01 |  | 184 | 1.65  (0.50 - 5.49) | 4.15E-01 |  |
|  | Egger  intercept |  | 1.01  (0.99 - 1.02) | 6.59E-01 |  |  | 1.01  (0.99 - 1.03) | 3.86E-01 |  |  | 1.01  (0.99 - 1.04) | 3.60E-01 |  |  | 1.01  (0.99 - 1.04) | 3.26E-01 |  |
| T2DM | MR Egger | 159 | 1.09  (0.96 - 1.24) | 1.99E-01 | 9.26E-01 | 159 | 1.01  (0.83 - 1.24) | 8.93E-01 | 9.18E-01 | 157 | 1.21  (0.94 - 1.55) | 1.37E-01 | 9.67E-01 | 159 | 1.100  (0.86 - 1.41) | 4.54E-01 | 3.69E-01 |
|  | Weighted  median | 159 | 1.03  (0.91 - 1.16) | 6.78E-01 |  | 159 | 1.08  (0.91 - 1.29) | 3.56E-01 |  | 157 | 1.02  (0.82 - 1.27) | 8.31E-01 |  | 159 | 0.94  (0.76 - 1.16) | 5.61E-01 |  |
|  | IVW | 159 | 1.05  (0.98 - 1.11) | 1.68E-01 | 9.30E-01 | 159 | 1.03  (0.93 - 1.13) | 6.07E-01 | 9.26E-01 | 157 | 1.07  (0.95 - 1.20) | 2.86E-01 | 9.66E-01 | 159 | 1.05  (0.94 - 1.19) | 3.80E-01 | 3.87E-01 |
|  | Simple  mode | 159 | 1.09  (0.87 - 1.36) | 4.45E-01 |  | 159 | 1.10  (0.79 - 1.54) | 5.69E-01 |  | 157 | 1.06  (0.70 - 1.60) | 7.81E-01 |  | 159 | 0.71  (0.45 - 1.11) | 1.31E-01 |  |
|  | Weighted  mode | 159 | 1.06  (0.94 - 1.19) | 3.24E-01 |  | 159 | 1.05  (0.87 - 1.26) | 6.11E-01 |  | 157 | 1.06  (0.84 - 1.34) | 6.26E-01 |  | 159 | 0.98  (0.78 - 1.23) | 8.53E-01 |  |
|  | Egger  intercept |  | 1.00  (0.99 - 1.01) | 4.76E-01 |  |  | 1.00  (0.99 - 1.01) | 8.96E-01 |  |  | 0.99  (0.97 - 1.01) | 2.64E-01 |  |  | 1.00  (0.98 - 1.01) | 7.05E-01 |  |
| ^a^ Only independent SNPs (*r^2^* < 0.001 within 10,000 kb windows), strongly associated (*P* ≤ 5E-08) were used as genetic instruments for the exposure.  ^b^ The odds ratios (OR) correspond to a 1 standard deviation increase for continuous traits (BMI, smoking, LDL-C, and SBP) and as per unit increase in the log odds ratio for genetically proxied T2DM liability.  Abbreviations: BMI, body mass index; CI, confidence interval; IVW, inverse-variance weighted; LDL-C, low-density lipoprotein cholesterol; Michigan Genomics Initiative, MGI; N, number of SNPs used; SBP, systolic blood pressure; Smoking, Lifetime smoking index; T2DM, type 2 diabetes mellitus. | | | | | | | | | | | | | | | | | |

**SUPPLEMENTARY FIGURES**


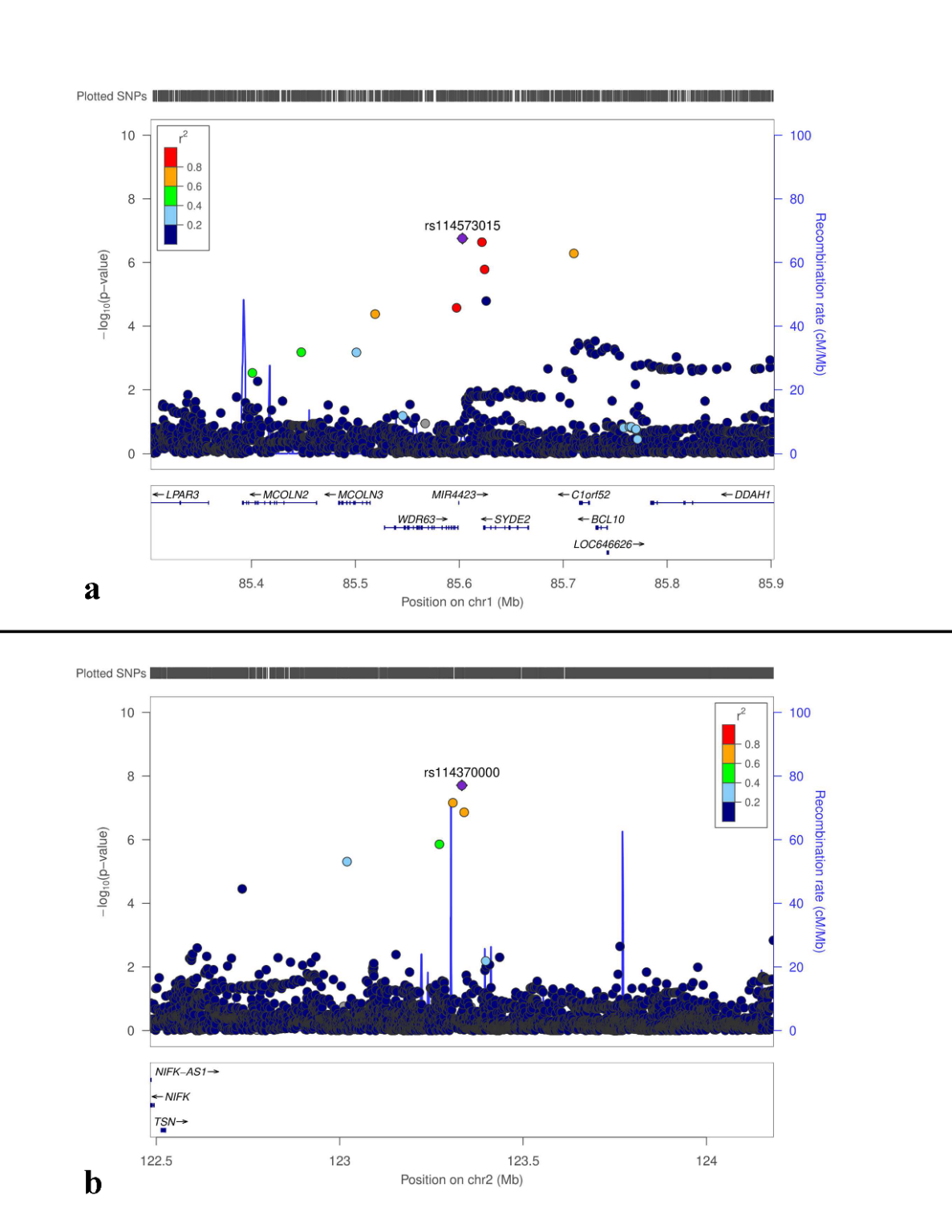
**Supplementary Figure 1.** Regional association plot for loci associated with the risk of upper urinary tract infection for the female-only analysis.

*Legend:* The *y*-axis represents the negative logarithm (base 10) of the variant *P* value (likelihood ratio test), and the *x*-axis represents the position on the chromosome, with the names and locations of genes and nearest genes shown at the bottom. The variants with the lowest *P* value in the region are marked by a purple diamond. The colors of the other variants indicate their *r*^2^ with the lead variant. The European population from the 1000 Genomes Project [9], November 2014 release, was used as reference, on genome build hg19. (a) The locus on chromosome 1 with index variant rs114573015. (b) The locus on chromosome 2 with index variant rs114370000.

**Supplementary Figure 2.** Regional association plot for loci associated with the risk of upper urinary tract infection for the male-only analysis.


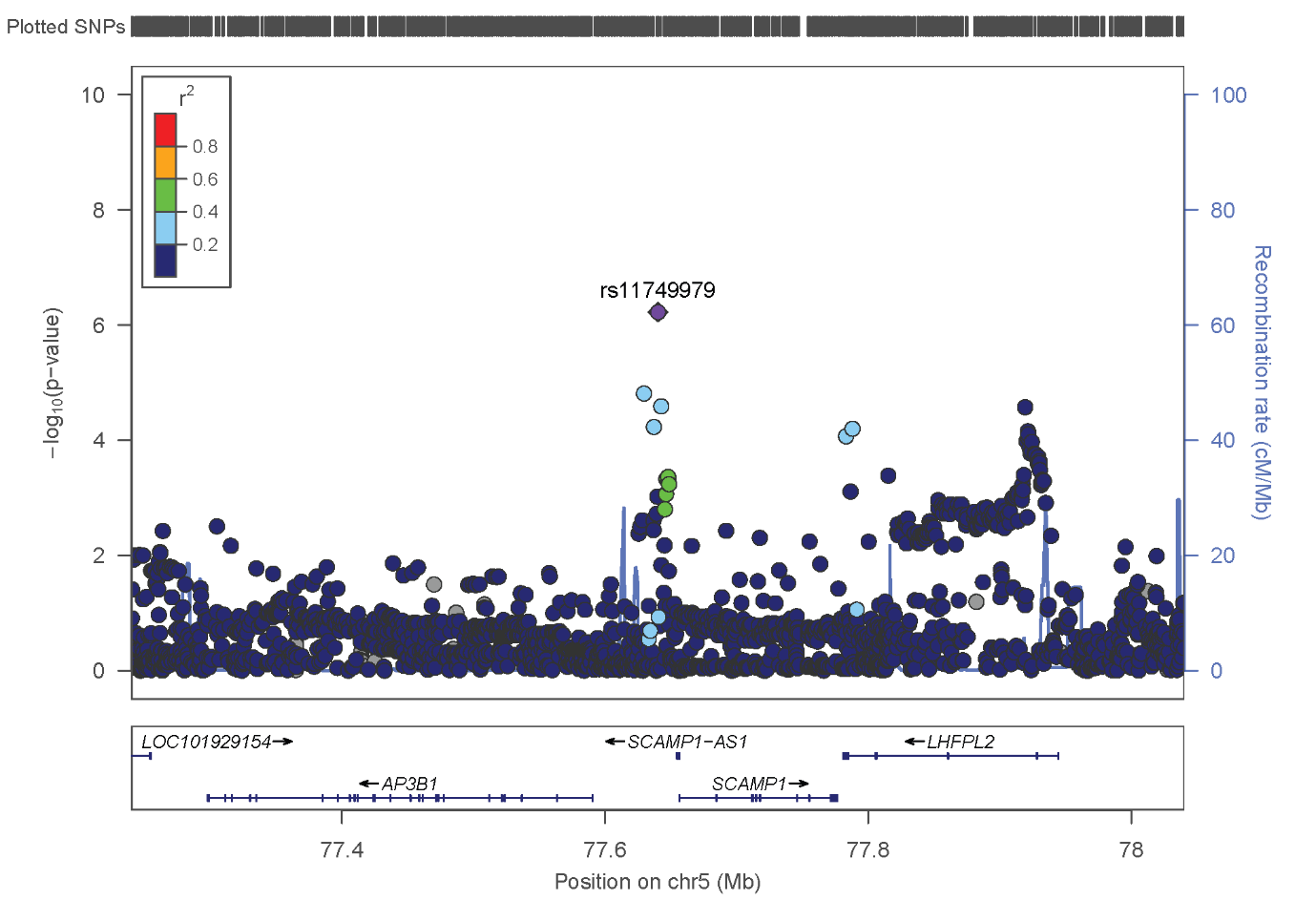


*Legend:* The *y*-axis represents the negative logarithm (base 10) of the variant *P* value (likelihood ratio test), and the *x*-axis represents the position on the chromosome, with the names and locations of genes and nearest genes shown at the bottom. The variant with the lowest *P* value in the region is marked by a purple diamond. The colors of the other variants indicate their *r*^2^ with the lead variant. The European population from the 1000 Genomes Project [9], November 2014 release, was used as reference, on genome build hg19. The locus on chromosome 5 with index variant rs11749979.


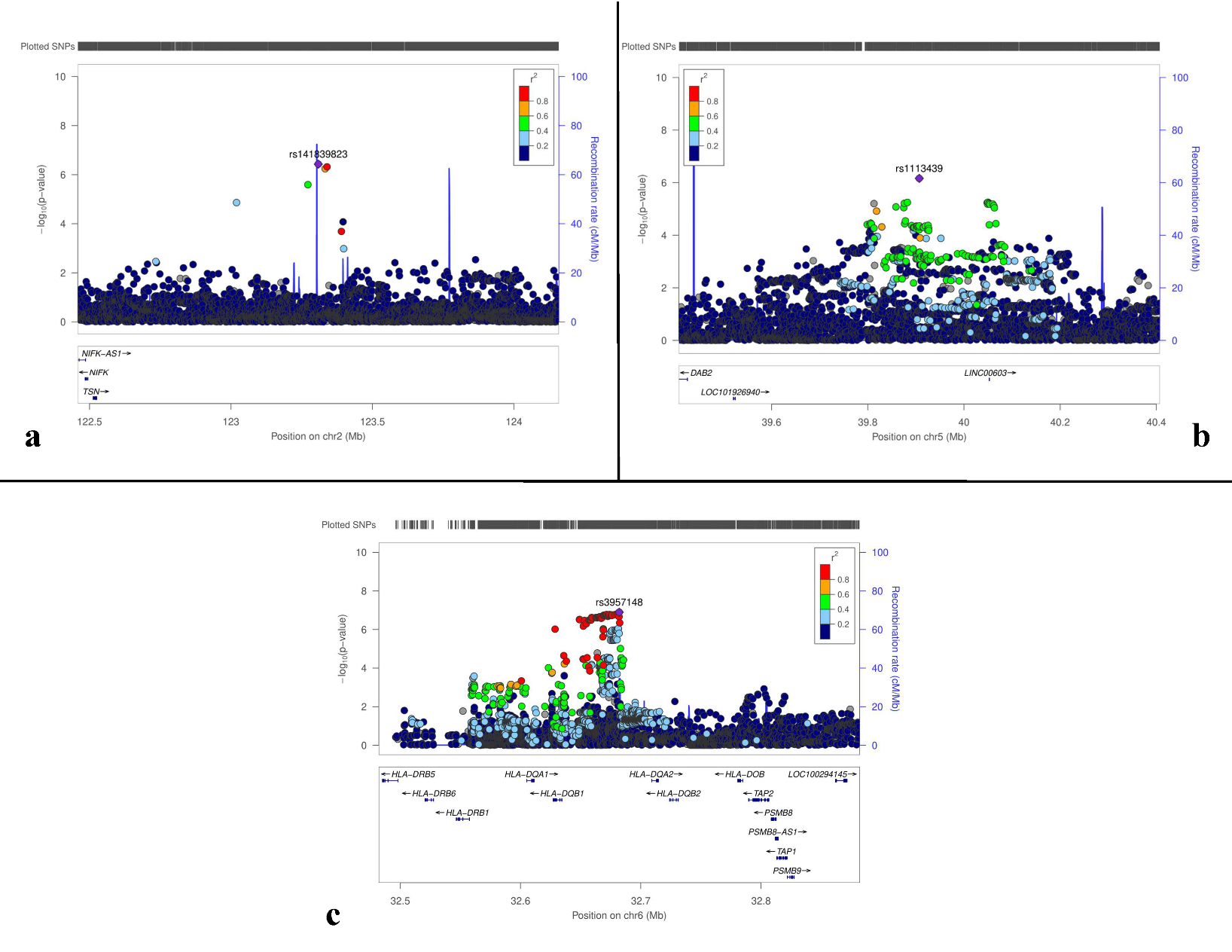
**Supplementary Figure 3.** Regional association plot for loci associated with the risk of upper urinary tract infection for the analysis of both sexes.

*Legend:* The *y*-axis represents the negative logarithm (base 10) of the variant *P* value (likelihood ratio test), and the *x*-axis represents the position on the chromosome, with the names and locations of genes and nearest genes shown at the bottom. The variants with the lowest *P* value in the region are marked by a purple diamond. The colors of the other variants indicate their *r*^2^ with the lead variant. The European population from the 1000 Genomes Project [9], November 2014 release, was used as reference, on genome build hg19. (a) The locus on chromosome 2 with index variant rs141839823. (b) The locus on chromosome 5 with index variant rs1113439. (c) the locus on chromosome 6 located in the HLA region with index variant rs3957148.

**Supplementary Figure 4.** Manhattan plot of the genome-wide association analysis for the risk of upper urinary tract infection in UK Biobank.


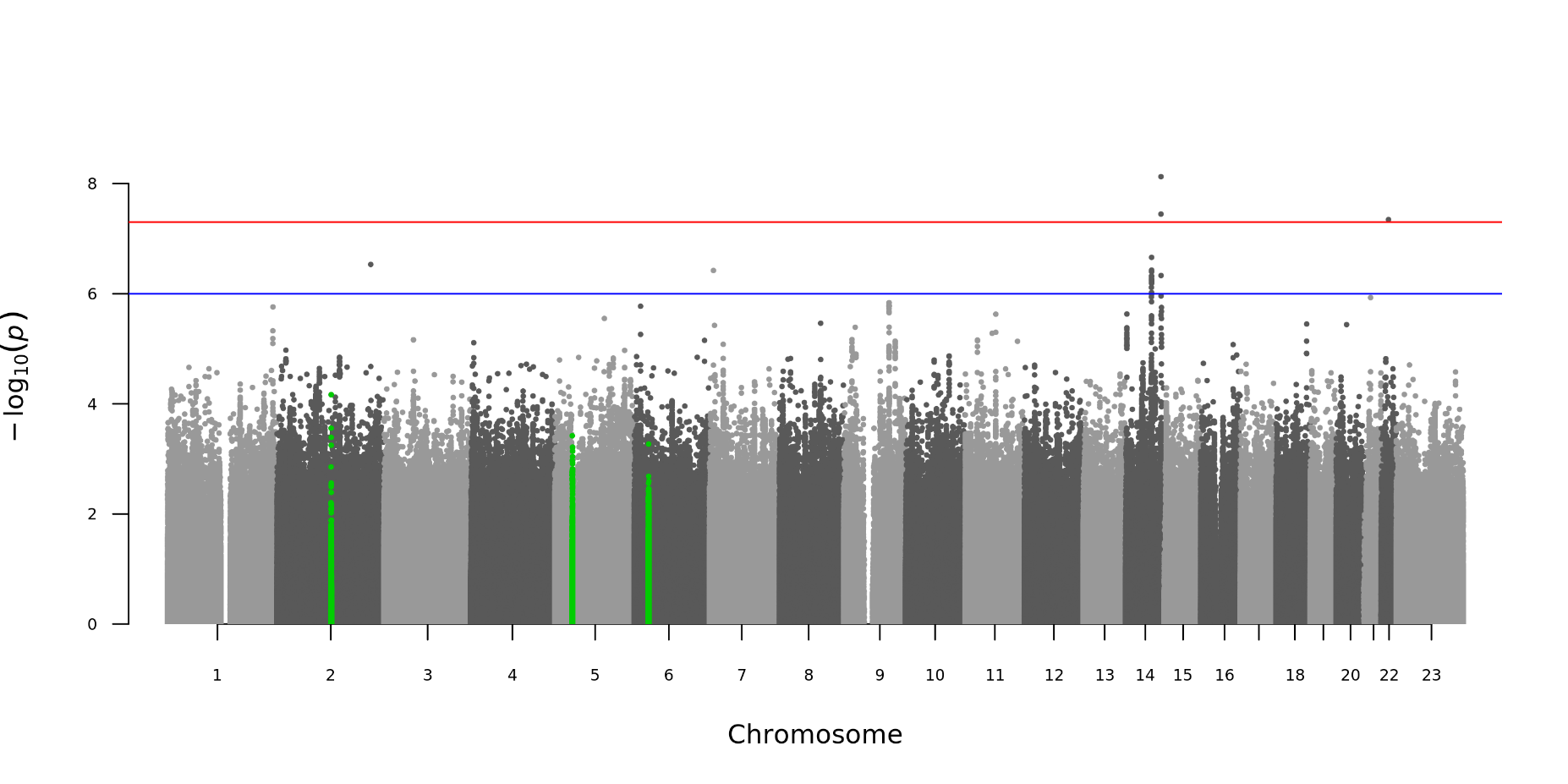


*Legend:* The *x*-axis shows the genomic position (chromosomes 1-23; where 23 is the X chromosome), and the *y*-axis represents the negative logarithm (base 10) of the variant *P* value. The blue line indicates the genome-wide suggestive threshold (*P* ≤ 1E-06), while the red line indicates genome-wide significant threshold (*P* ≤ 5E-08). The three genetic loci (±500 kb of lead variant) that reached the genome-wide suggestive threshold in the analysis of both sexes have been highlighted in green.

**Supplementary Figure 5.** Manhattan plot of the genome-wide association analysis for the risk of upper urinary tract infection in HUNT.


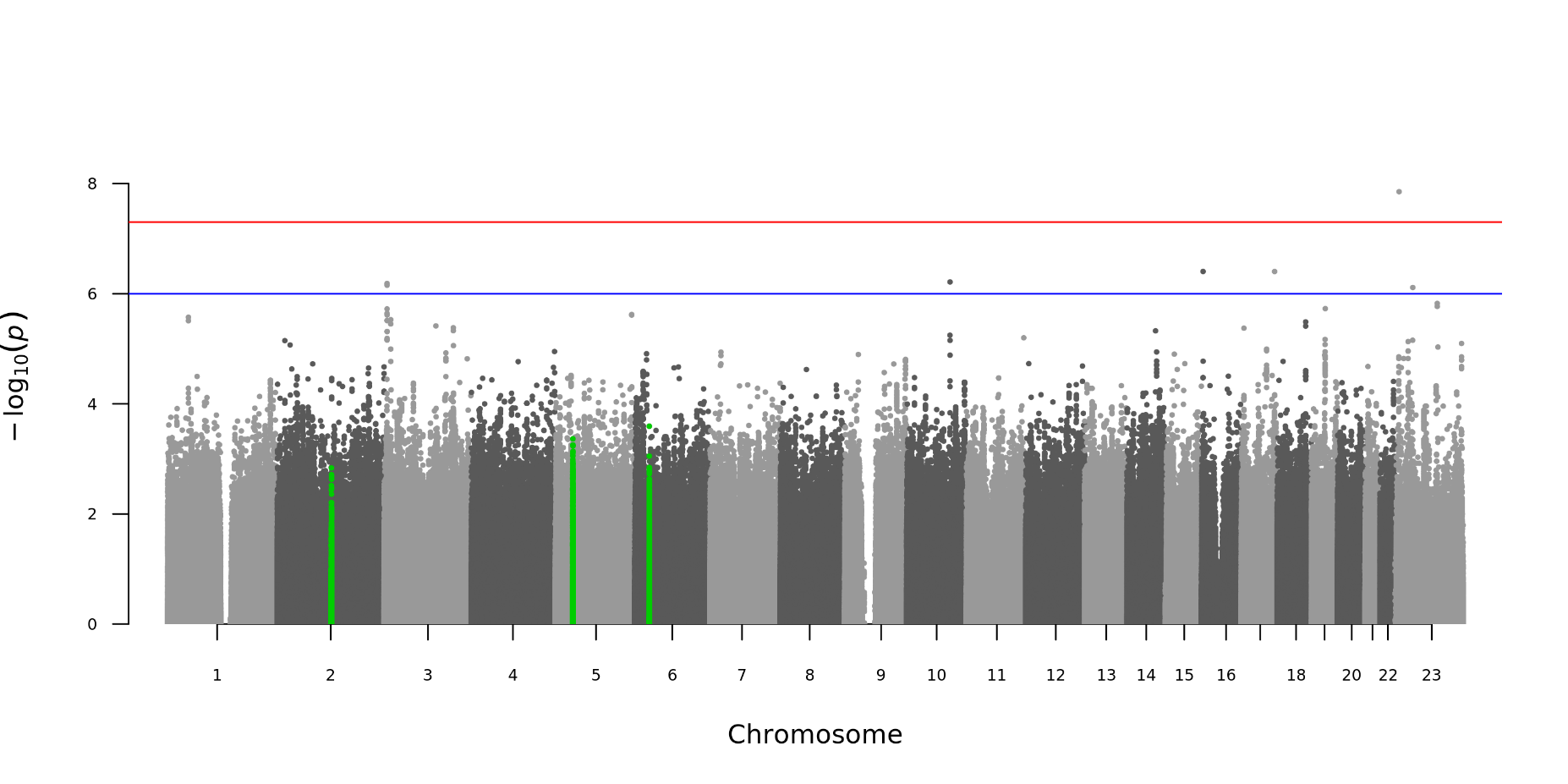


*Legend:* The *x*-axis shows the genomic position (chromosomes 1-23; where 23 is the X chromosome), and the *y*-axis represents the negative logarithm (base 10) of the variant *P* value. The blue line indicates the genome-wide suggestive threshold (*P* ≤ 1E-06), while the red line indicates genome-wide significant threshold (*P* ≤ 5E-08). The three genetic loci (±500 kb of lead variant) that reached the genome-wide suggestive threshold in the analysis of both sexes analysis have been highlighted in green.

**Supplementary Figure 6.** Manhattan plot of the genome-wide association analysis for the risk of upper urinary tract infection in Michigan Genomics Initiative.


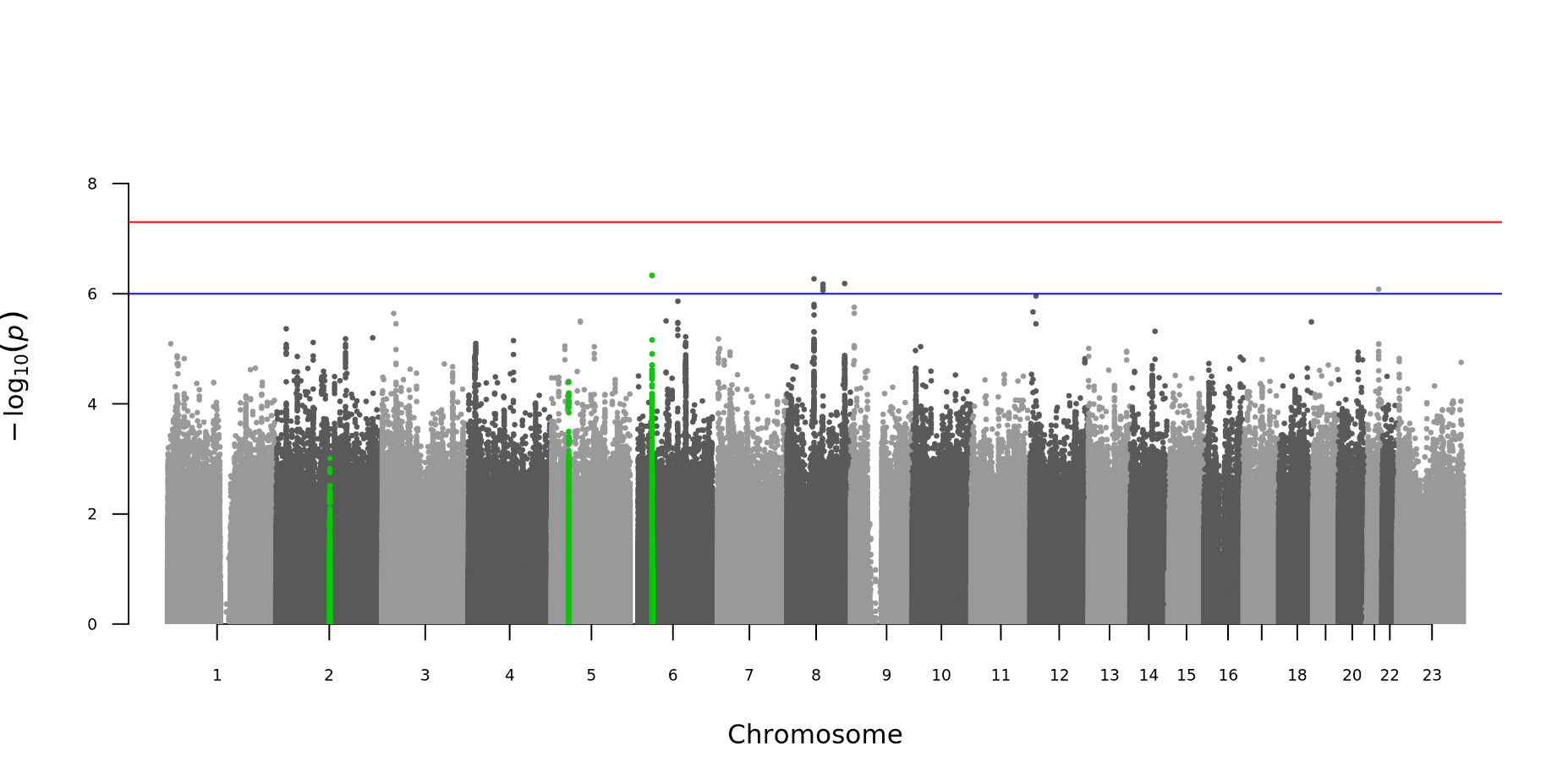


*Legend:* The *x*-axis shows the genomic position (chromosomes 1-23; where 23 is the X chromosome), and the *y*-axis represents the negative logarithm (base 10) of the variant *P* value. The blue line indicates the genome-wide suggestive threshold (*P* ≤ 1E-06), while the red line indicates genome-wide significant threshold (*P* ≤ 5E-08). The three genetic loci (±500 kb of lead variant) that reached the genome-wide suggestive threshold in the analysis of both sexes have been highlighted in green.

**Supplementary Figure 7.** Quantile-quantile for the female-only analysis.


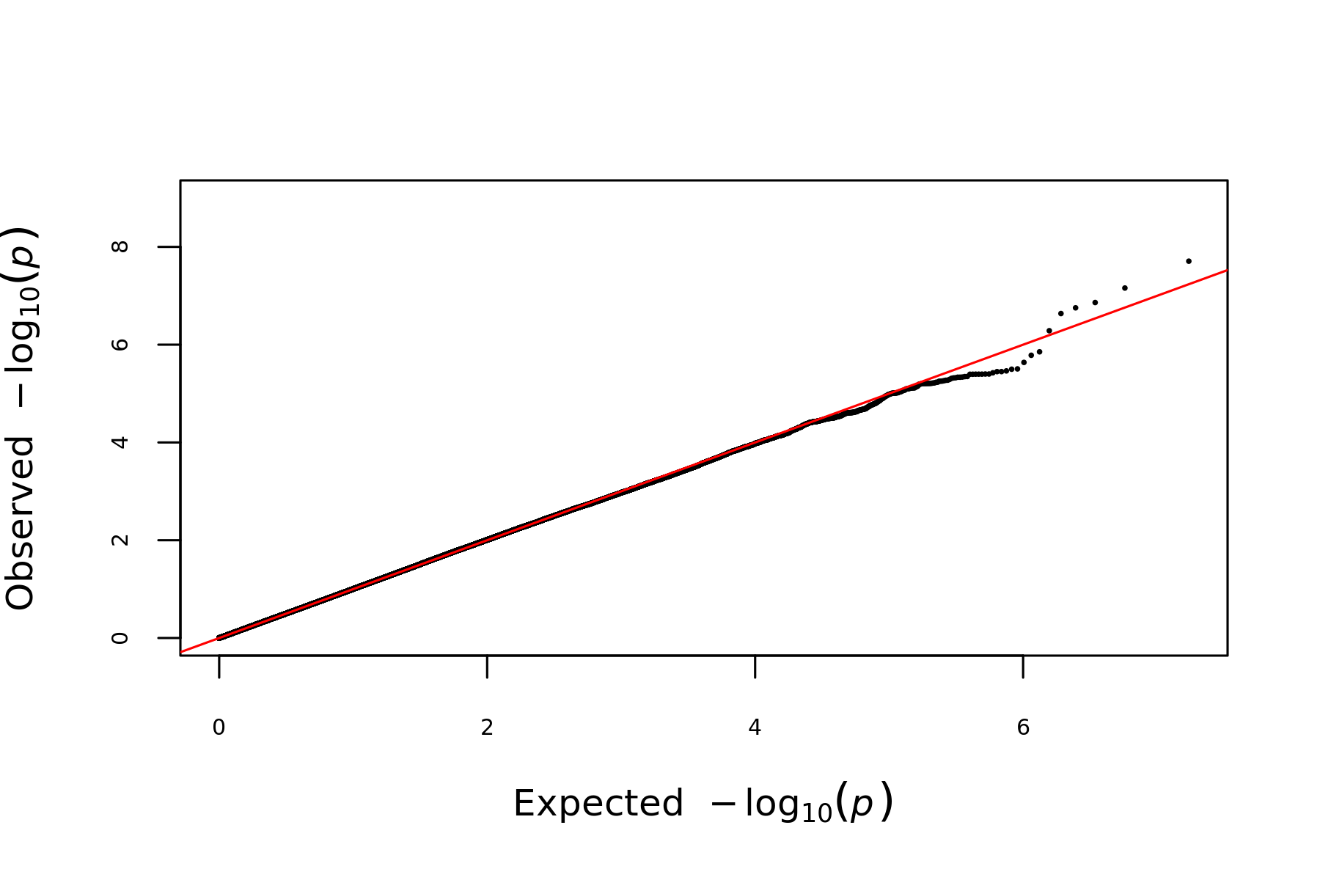


*Legend:* Quantile-quantile plot showing the results from the female-only meta-analysis. Axes display the observed (*y*-axis) and expected (*x*-axis) -log_10_ transformed *P* values. The black dots represent observed *P* values, while the red line represents expected *P* values under the null distribution.

**Supplementary Figure 8.** Quantile-quantile for male-only analysis.


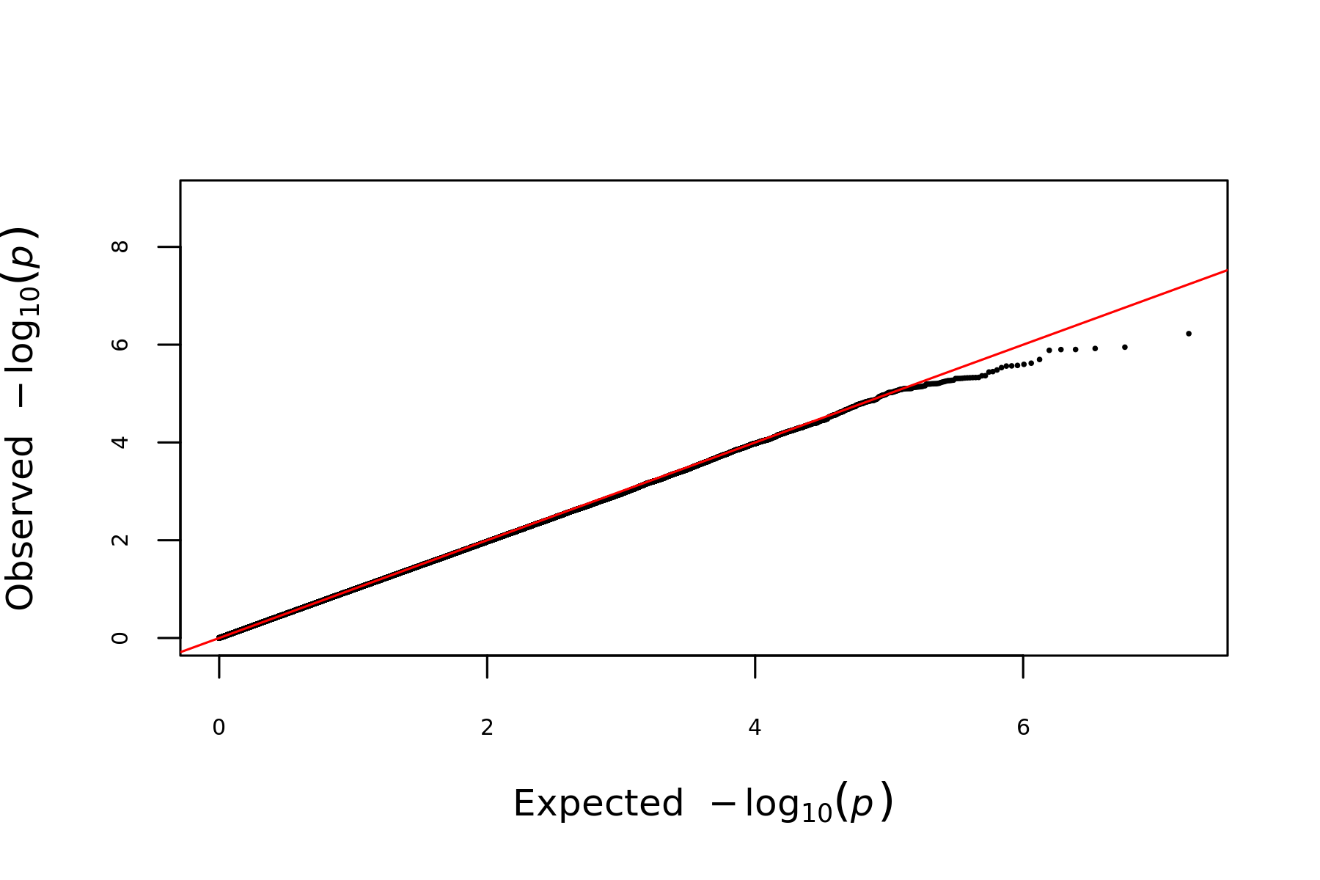


*Legend:* Quantile-quantile plot showing the results from the male-only analysis. Axes display the observed (*y*-axis) and expected (*x*-axis) -log_10_ transformed *P* values. The black dots represent observed *P* values, while the red line represents expected *P* values under the null distribution.

**Supplementary Figure 9.** Quantile-quantile for the meta-analysis of both sexes.


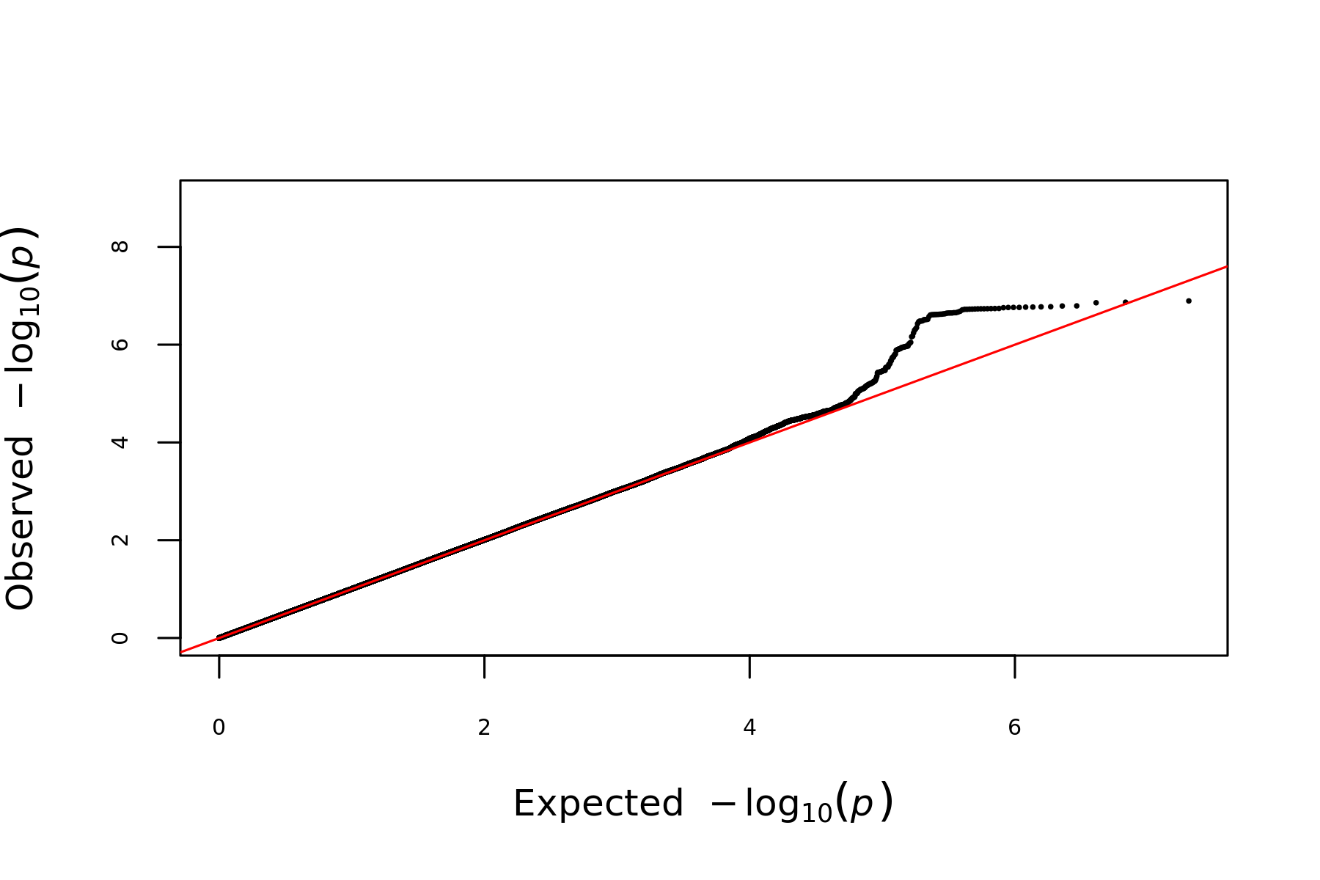


*Legend:* Quantile-quantile plot showing the results from the meta-analysis of both sexes using data from UK Biobank, HUNT, and MGI. Axes display the observed (*y*-axis) and expected (*x*-axis) -log_10_ transformed *P* values. The black dots represent observed *P* values, while the red line represents expected *P* values under the null distribution.

**Supplementary Figure 10.** Quantile-quantile for analysis of both sexes in UK Biobank.


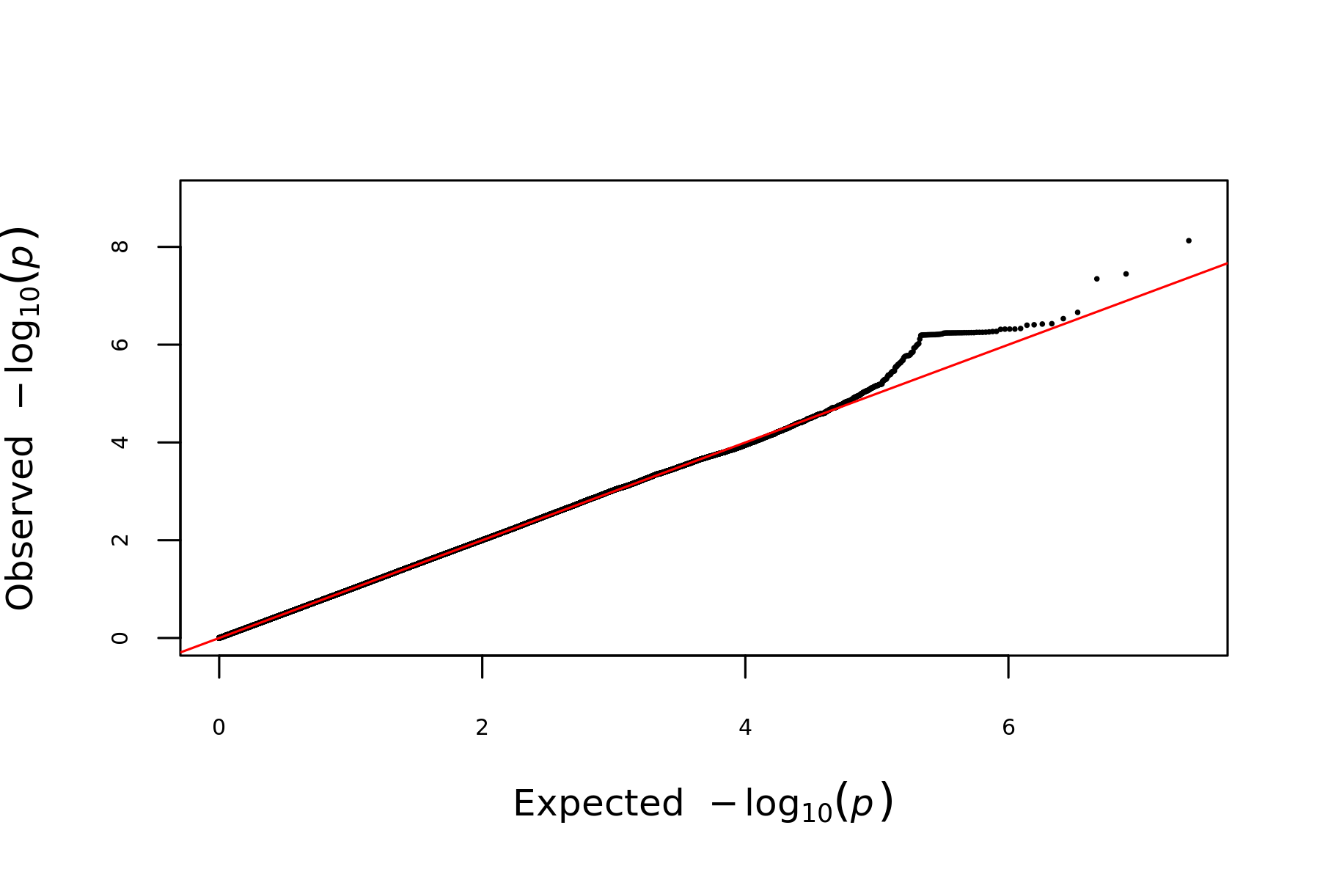


*Legend:* Axes display the observed (*y*-axis) and expected (*x*-axis) -log_10_ transformed *P* values. The black dots represent observed *P* values, while the red line represents expected *P* values under the null distribution.

**Supplementary Figure 11.** Quantile-quantile for the analysis of both sexes in HUNT.


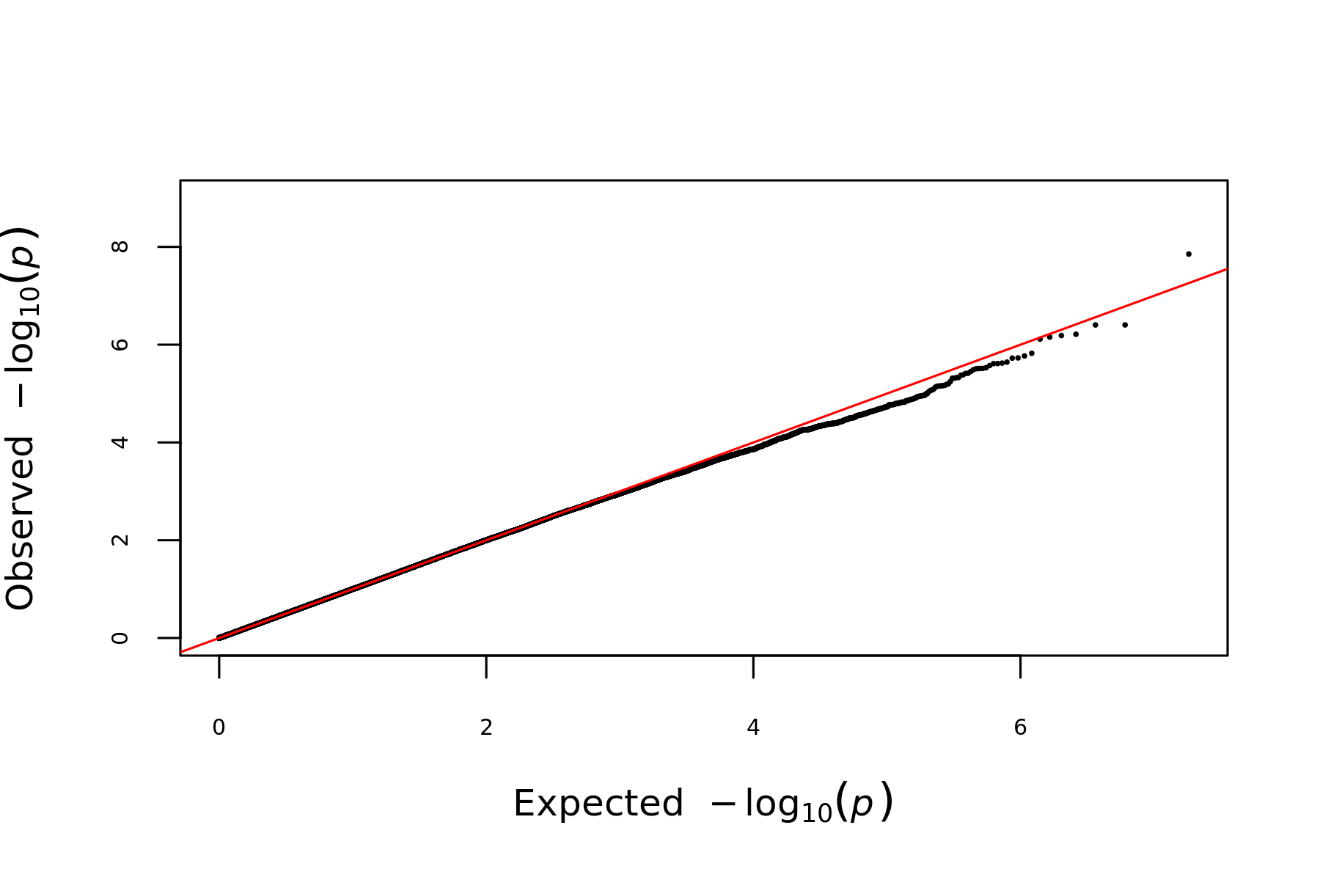


*Legend:* Axes display the observed (*y*-axis) and expected (*x*-axis) -log_10_ transformed *P* values. The black dots represent observed *P* values, while the red line represents expected *P* values under the null distribution.

**Supplementary Figure 12.** Quantile-quantile for the analysis of both sexes in Michigan Genomics Initiative.


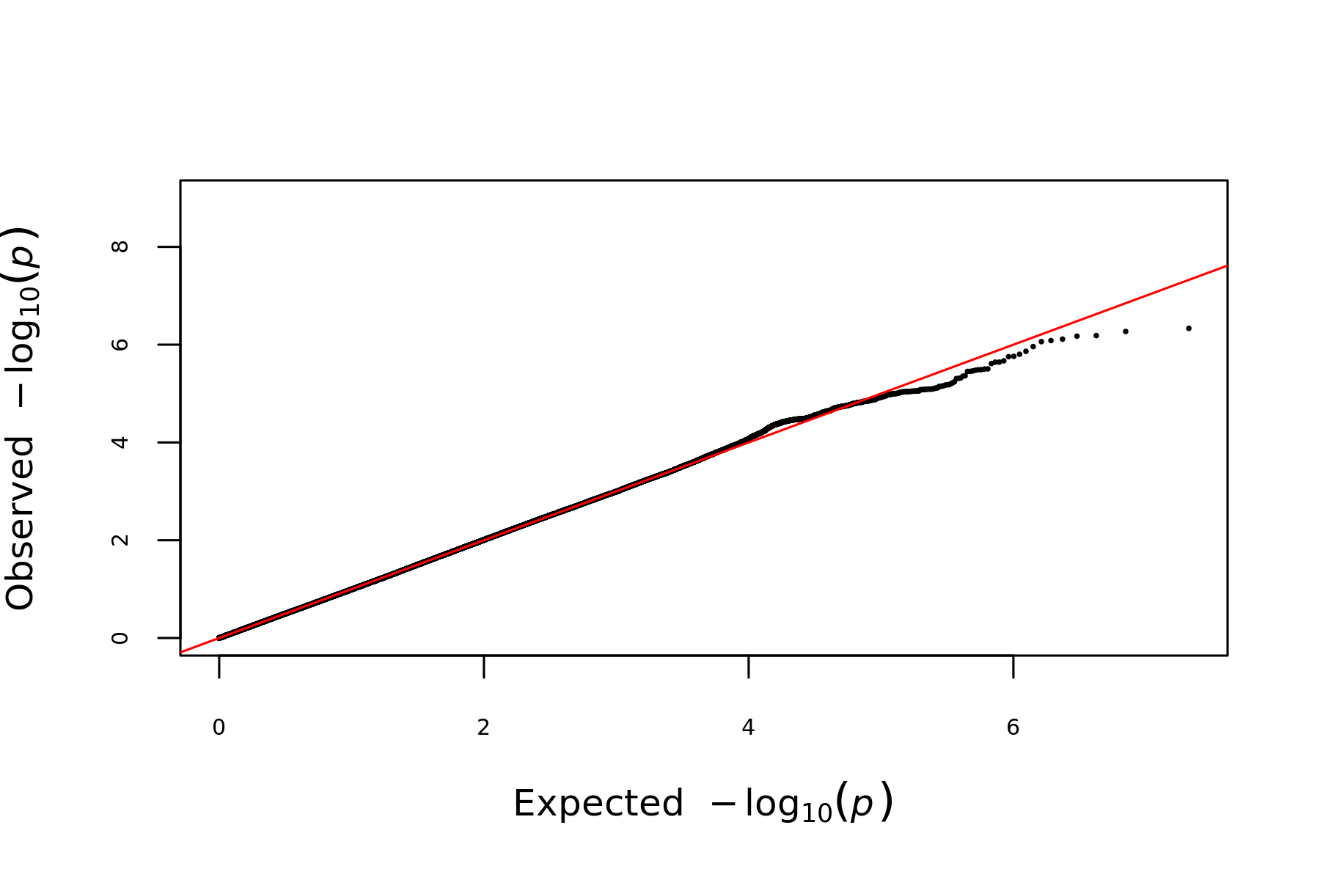


*Legend:* Axes display the observed (*y*-axis) and expected (*x*-axis) -log_10_ transformed *P* values. The black dots represent observed *P* values, while the red line represents expected *P* values under the null distribution.

**Supplementary Figure 13.** Scatter plot of MR analysis on lifetime smoking index and upper UTI of the female-only analysis.


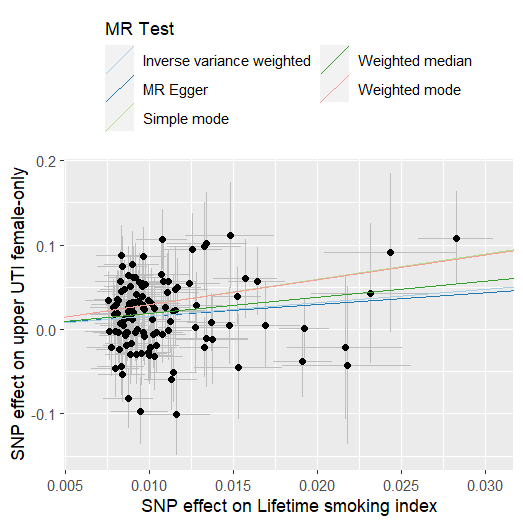


*Legend:* Scatter plot of the MR-analysis (*r^2^* < 0.001 within 10,000 kb windows and *P* ≤ 5E-08) using lifetime smoking index as exposure and the female-only meta-analysis as outcome.

**Supplementary Figure 14.** Leave one out plot of MR analysis on lifetime smoking index and upper UTI of the female-only analysis.


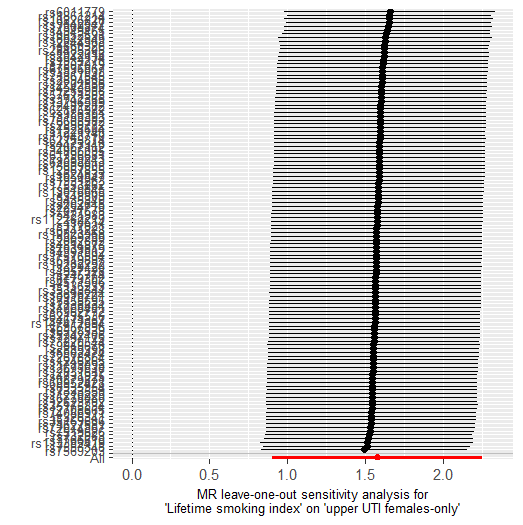


*Legend:* Leave-one-out plot of the MR-analysis (*r^2^* < 0.001 within 10,000 kb windows and *P* ≤ 5E-08) using lifetime smoking index as exposure and the female-only meta-analysis as outcome.**Supplementary Figure 15.** Scatter plot of MR analysis on lifetime smoking index and upper UTI in both sexes.


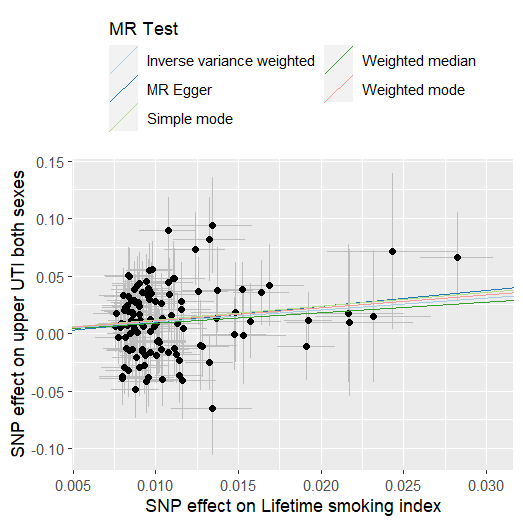


*Legend:* Scatter plot of the MR-analysis (*r^2^* < 0.001 within 10,000 kb windows and *P* ≤ 5E-08) using lifetime smoking index as exposure and the analysis conducted on both sexes as outcome.

**Supplementary Figure 16.** Leave one out plot of MR analysis on lifetime smoking index and upper UTI of both sexes.


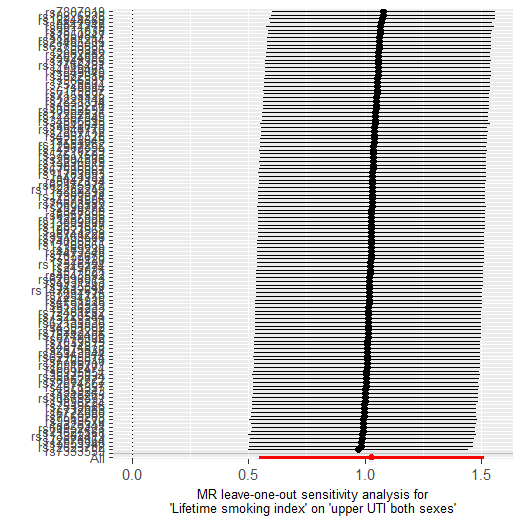


*Legend:* Leave-one-out plot of the MR-analysis (*r^2^* < 0.001 within 10,000 kb windows and *P* ≤ 5E-08) using lifetime smoking index as exposure and the analysis of both sexes as outcome.

**Supplementary Figure 17.** Scatter plot of MR analysis on systolic blood pressure and upper UTI of the male-only analysis.


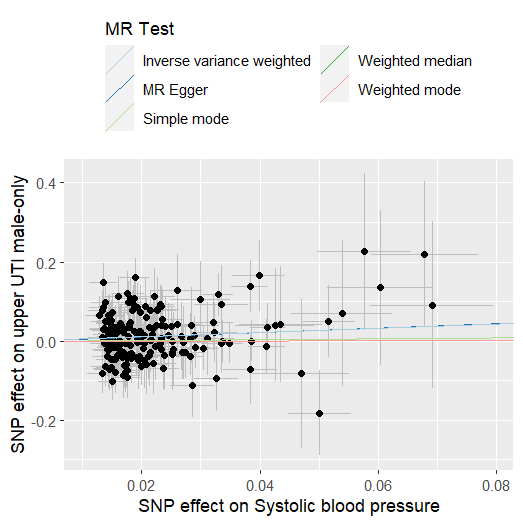


*Legend:* Scatter plot of the MR-analysis (*r^2^* < 0.001 within 10,000 kb windows and *P* ≤ 5E-08) using systolic blood pressure as exposure and the male-only meta-analysis as outcome.

**Supplementary Figure 18.** Leave one out plot of MR analysis on systolic blood pressure index and upper UTI of the male-only analysis.


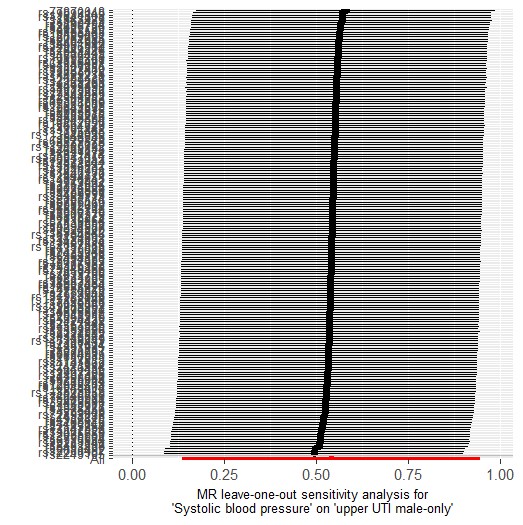


*Legend:* Leave-one-out plot of the MR-analysis (*r^2^* < 0.001 within 10,000 kb windows and *P* ≤ 5E-08) using systolic blood pressure as exposure and the male-only meta-analysis as outcome.

**REFERENCES**

1. Bycroft C, Freeman C, Petkova D, et al. The UK Biobank resource with deep phenotyping and genomic data. Nature **2018**; 562:203-9.

2. Sudlow C, Gallacher J, Allen N, et al. UK biobank: an open access resource for identifying the causes of a wide range of complex diseases of middle and old age. PLoS Med **2015**; 12:e1001779.

3. Brumpton BM, Graham S, Surakka I, et al. The HUNT Study: a population-based cohort for genetic research. Cell Genomics **2022**; 2:100193.

4. Krokstad S, Langhammer A, Hveem K, et al. Cohort profile: the HUNT study, Norway. Int J Epidemiol **2013**; 42:968-77.

5. Holmen J, Midthjell K, Krüger Ø, et al. The Nord-Trøndelag Health Study 1995–97 (HUNT 2): objectives, contents, methods and participation. Norsk epidemiologi **2003**; 13:19-32.

6. Zawistowski M, Fritsche LG, Pandit A, et al. The Michigan Genomics Initiative: A biobank linking genotypes and electronic clinical records in Michigan Medicine patients. Cell Genom **2023**; 3:100257.

7. O'Connell J, Sharp K, Shrine N, et al. Haplotype estimation for biobank-scale data sets. Nat Genet **2016**; 48:817-20.

8. UK10K consortium. The UK10K project identifies rare variants in health and disease. Nature **2015**; 526:82-90.

9. Auton A, Brooks LD, Durbin RM, et al. A global reference for human genetic variation. Nature **2015**; 526:68-74.

10. Howie B, Fuchsberger C, Stephens M, Marchini J, Abecasis GR. Fast and accurate genotype imputation in genome-wide association studies through pre-phasing. Nat Genet **2012**; 44:955-9.

11. Ferreira MA, Vonk JM, Baurecht H, et al. Shared genetic origin of asthma, hay fever and eczema elucidates allergic disease biology. Nat Genet **2017**; 49:1752-7.

12. Jun G, Flickinger M, Hetrick KN, et al. Detecting and estimating contamination of human DNA samples in sequencing and array-based genotype data. Am J Hum Genet **2012**; 91:839-48.

13. Loh P-R, Danecek P, Palamara PF, et al. Reference-based phasing using the Haplotype Reference Consortium panel. Nat Genet **2016**; 48:1443.

14. Loh P-R. BOLT-LMM v2. 3.2 user manual. Available at: <https://alkesgroup.broadinstitute.org/BOLT-LMM/downloads/old/BOLT-LMM_v2.3.2_manual.pdf>. 2019.

15. Kretzschmar W, Mahajan A, Sharp K, McCarthy M, Consortium HR. A reference panel of 64,976 haplotypes for genotype imputation. Nat Genet **2016**; 48.

16. Taliun D, Harris DN, Kessler MD, et al. Sequencing of 53,831 diverse genomes from the NHLBI TOPMed Program. Nature **2021**; 590:290-9.

17. Zhou W, Nielsen JB, Fritsche LG, et al. Efficiently controlling for case-control imbalance and sample relatedness in large-scale genetic association studies. Nat Genet **2018**; 50:1335-41.

18. Loh P-R, Tucker G, Bulik-Sullivan BK, et al. Efficient Bayesian mixed-model analysis increases association power in large cohorts. Nat Genet **2015**; 47:284.

19. Hinrichs AS, Karolchik D, Baertsch R, et al. The UCSC genome browser database: update 2006. Nucleic Acids Res **2006**; 34:D590-D8.

20. Hemani G, Zheng J, Elsworth B, et al. The MR-Base platform supports systematic causal inference across the human phenome. Elife **2018**; 7:e34408.

21. Yengo L, Sidorenko J, Kemper KE, et al. Meta-analysis of genome-wide association studies for height and body mass index in∼ 700000 individuals of European ancestry. Hum Mol Genet **2018**; 27:3641-9.

22. Locke AE, Kahali B, Berndt SI, et al. Genetic studies of body mass index yield new insights for obesity biology. Nature **2015**; 518:197-206.

23. Wootton RE, Richmond RC, Stuijfzand BG, et al. Evidence for causal effects of lifetime smoking on risk for depression and schizophrenia: a Mendelian randomisation study. Psychol Med **2019**; 50:2435-43.

24. Willer CJ, Schmidt EM, Sengupta S, et al. Discovery and refinement of loci associated with lipid levels. Nat Genet **2013**; 45:1274.

25. Carter AR, Gill D, Davies NM, et al. Understanding the consequences of education inequality on cardiovascular disease: mendelian randomisation study. BMJ **2019**; 365:l1855.

26. Mahajan A, Taliun D, Thurner M, et al. Fine-mapping type 2 diabetes loci to single-variant resolution using high-density imputation and islet-specific epigenome maps. Nat Genet **2018**; 50:1505-13.

27. Tängdén T, Gustafsson S, Rao AS, Ingelsson E. A genome-wide association study in a large community-based cohort identifies multiple loci associated with susceptibility to bacterial and viral infections. Sci Rep **2022**; 12:1-14.

28. Tian C, Hromatka BS, Kiefer AK, et al. Genome-wide association and HLA region fine-mapping studies identify susceptibility loci for multiple common infections. Nat Commun **2017**; 8:599.

29. Sakaue S, Kanai M, Tanigawa Y, et al. A cross-population atlas of genetic associations for 220 human phenotypes. Nat Genet **2021**; 53:1415-24.
